# ERM Inhibition Confers Ferroptosis Resistance through ROS‐Induced NRF2 Signaling

**DOI:** 10.1002/advs.202513310

**Published:** 2026-01-27

**Authors:** Menghao Qiao, Liqun Zhou, Minhua Zhou, Yu Fang, Haiying Mai, Lingbo Cao, Kun Xu, Yuan Sang, Minyi Chen, Jiewei Huang, Peiyi Huang, Zhipeng Yan, Chao Wang, Zhangshuai Dai, Dichun Huang, Ronghan He, Lijuan Pang, Yunmiao Guo, Ting Gang Chew, Junqi Huang

**Affiliations:** ^1^ Key Laboratory of Regenerative Medicine of Ministry of Education Institute of Aging and Regenerative Medicine Department of Developmental & Regenerative Medicine College of Life Science and Technology Jinan University Guangzhou China; ^2^ Department of Cardiology of the Second Affiliated Hospital Zhejiang University School of Medicine Zhejiang University Hangzhou China; ^3^ Zhejiang University‐University of Edinburgh Institute Zhejiang University Hangzhou China; ^4^ Department of Joint and Trauma Surgery The Third Affiliated Hospital of Sun Yat‐Sen University Guangzhou China; ^5^ Department of Pathology Central People's Hospital of Zhanjiang Guangdong Medical University Zhanjiang Central Hospital Zhanjiang China; ^6^ Zhanjiang Institute of Clinical Medicine Central People's Hospital of Zhanjiang Guangdong Medical University Zhanjiang Central Hospital Zhanjiang China

**Keywords:** Actin, ERM proteins, Ferroptosis, HMOX1, NRF2, ROS

## Abstract

Ferroptosis is an iron‐dependent form of programmed cell death governed by redox homeostasis. Although Ezrin, Radixin, and Moesin (ERM) proteins are established membrane‐actin cytoskeleton linkers, their role in ferroptosis remains unexplored. Here, ERM proteins are identified as modulators of erastin‐induced ferroptosis. In human fibrosarcoma HT‐1080 cells, pharmacological inhibition of ERM phosphorylation, knockdown of individual ERM members, or overexpression of a phospho‐deficient Ezrin mutant (T567A) consistently attenuated ferroptosis, whereas wild‐type ERM overexpression enhances ferroptosis susceptibility. Mechanistically, ERM inhibition leads to F‐actin depolymerization accompanied by a modest rise in reactive oxygen species (ROS). F‐actin stabilization prevents this ROS surge and restores ferroptotic sensitivity, whereas its depolymerization mimics the protective effect of ERM inhibition. ROS elevation triggers KEAP1 degradation, stabilizing NRF2 and promoting its nuclear translocation. Activated nuclear NRF2 induces antioxidant genes, particularly *HMOX1*, a key effector of heme catabolism that enhances redox buffering and limits lipid peroxidation, ultimately conferring resistance to ferroptosis. The protective effects of ERM inhibition are further validated in ferroptosis‐relevant ex vivo and in vivo models. Notably, other pro‐oxidants similarly attenuate ferroptosis at appropriate concentrations. Together, these results establish ERM proteins as regulators of ferroptosis and reveal an underappreciated group of ferroptosis inhibitors that engage ROS‐NRF2‐mediated redox‐adaptation.

## Graphical abstract

ERM inhibition disrupts ERM‐actin interactions, elevating ROS and triggering KEAP1 degradation, which stabilizes and activates NRF2. Nuclear NRF2 induces cytoprotective genes, notably HMOX1, enhancing redox buffering and suppressing lipid peroxidation to resist erastin‐induced ferroptosis. ERM inhibitors thus act as pro‐oxidants that paradoxically promote ferroptosis resistance, with protective effects observed in ex vivo and in vivo models.

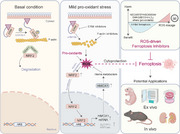

## 1 Introduction

Ferroptosis, a form of programmed cell death distinguished by iron‐dependent lipid peroxidation, has garnered significant attention since its inception by the Stockwell group in 2012 [1]. Ferroptosis plays multifaceted roles in various physiological and pathological contexts, exerting profound effects in cancer, neurodegenerative diseases, and ischemic organ injuries such as ischemic brain disease, acute kidney failure, and liver damage [2, 3, 4, 5, 6, 7, 8, 9, 10]. Central to the process of ferroptosis is the intricate interplay between toxic ROS generation and lipid peroxidation (Lipid ROS) accumulation, and is governed by a network of proteins and compounds involved in antioxidant systems, lipid metabolism, and ion homeostasis [11, 12, 13, 14, 15]. Key players in this complex regulatory network include but not limited to iron (whose levels delicately influence the level of ROS and lipid ROS), system Xc^−^ (a cystine‐glutamate antiporter essential for maintaining cellular redox balance), and GPX4 (glutathione peroxidase 4, a selenoprotein crucial for neutralizing lipid peroxides). Furthermore, emerging research sheds light on other antioxidant systems such as FSP1, DHODH, vitamin K, Coenzyme Q10, sex hormones, 7‐DHC, and GSTP1, each contributing a unique facet to the intricate regulation of ferroptosis [16, 17, 18, 19, 20, 21, 22].

The ERM family proteins, Ezrin, Radixin, and Moesin, are highly conserved plasma membrane‐associated proteins involved in cancer metastasis, Alzheimer's disease, cholestasis, and rare diseases such as Behcet's disease, X‐linked Moesin‐associated immunodeficiency [23, 24, 25, 26, 27, 28]. Mutations in the ERM proteins cause severe phenotypes in human and rodent [29, 30]. At the cellular level, ERM proteins are integral to various cellular processes such as cell migration, adhesion, morphogenesis, tumorigenesis, and immunological synapse formation [31, 32, 33]. The ERM proteins also bridge signal transduction pathways such as PI3K/AKT/mTOR, EGFR signaling, estrogen‐mediated signaling, and Hedgehog signaling [31, 34, 35, 36]. Their protein structure typically includes an N‐terminal membrane‐associated FERM domain, a central α‐helical region, and a C‐terminal ERM‐association domain (ERMAD). The ERM proteins switch between an open and closed conformation, which represents the active and inactive state respectively. The cytoplasmic ERM proteins are in a dormant closed conformation. Phosphorylation at Thr567 in Ezrin (corresponding to T564 in Radixin and T558 in Moesin) by kinases such as lymphocyte‐oriented kinase (LOK) and STE20‐like serine/threonine‐protein kinase (SLK) is a key step in activating the ERM proteins [37, 38, 39]. This phosphorylation diminishes the affinity between the N‐terminal FERM domain and the C‐ERMAD region, breaks their head‐to‐tail associations, and relocates ERM proteins to the plasma membrane [31, 40, 41]. Consequently, this phosphorylation reveals previously concealed binding sites on these domains for association with the actin cytoskeleton and other membrane‐associated proteins such as CD43/44, NHE1, and ICAM‐2 [41, 42, 43, 44]. Apart from the actin cytoskeleton, the ERM proteins interact with a variety of other proteins [34, 45, 46]. The binding of ERM proteins to actin filaments in turn can stabilize the actin cytoskeleton [31]. Importantly, high expression of ERM proteins is associated with metastasis and poor prognosis in certain cancer types [41, 47]. Thus, ERM proteins inhibition is believed to have prognostic implications in preventing cancer metastasis in patients. Inhibition of ERM proteins, particularly through small molecule inhibitors targeting ERM phosphorylation like NSC305787 and NSC668394, holds promise in anti‐cancer therapy and may have prognostic implications for preventing cancer metastasis [48, 49, 50, 51, 52]. While both chemicals showed no obvious toxic effect in murine models, NSC305787 exhibited a longer plasma half‐life than NSC668394^52^. Surprisingly, even though the ERM proteins are proved to be widely associated with cancer cell metastasis, their inhibitors have shown anti‐metastatic effects only in limited types of cancer. Previous studies have also shown that ERM proteins participate in programmed cell death processes, such as apoptosis and entosis [53]. Nevertheless, the role of the ERM proteins in ferroptosis remains unexplored [53].

ERM proteins link the plasma membrane to the underlying actin cytoskeleton, a dynamic network composed of globular and filamentous (F‐actin) forms. Together with actin‐associated proteins, this network is essential for numerous cellular functions, including cell division, migration, adhesion, intracellular trafficking, and morphogenesis [54, 55, 56, 57]. Actin polymerization is orchestrated by nucleation‐promoting factors such as FMN1, DIAPH1, DIAPH3, INF2, and the Arp2/3 complex. Beyond its structural roles, the actin cytoskeleton exhibits a bidirectional relationship with ROS [58]. On one hand, ROS can disrupt actin dynamics by oxidizing cysteine residues. On the other, actin can regulate ROS production by interacting with and facilitating the activation of NADPH oxidase (NOX) complexes, which are important sources of ROS [59]. Emerging evidence also points to a tight interplay between the actin cytoskeleton and programmed cell death pathways [53, 60]. Notably, the actin cytoskeleton has been implicated in ferroptosis regulation. In HeLa cells, treatment with Cytochalasin D, an actin polymerization inhibitor, appears to regulate ferroptosis susceptibility [61]. However, in Magnaporthe oryzae, the application of Cytochalasin E, another potent actin polymerization inhibitor, was shown to suppress ferroptosis [62]. Despite these intriguing connections and discrepancies, the molecular mechanisms linking actin cytoskeleton to ferroptosis remain poorly understood.

Nuclear factor erythroid 2‐related factor 2 (NRF2) is a master transcriptional regulator of cellular antioxidant responses. Under homeostatic conditions, NRF2 is tightly regulated by Kelch‐like ECH‐associated protein 1 (KEAP1), an adaptor protein for the Cullin3‐based E3 ubiquitin ligase complex [63]. KEAP1 binds NRF2 in a 2:1 stoichiometry, targeting it for proteasomal degradation and maintaining low NRF2 levels under normoxic, non‐stressed conditions [64]. Upon exposure to oxidative stress, oxidation of cysteine residues on KEAP1 disrupts its repressive interaction with NRF2 [65]. This leads to KEAP1 degradation, enabling NRF2 stabilization, nuclear translocation, and activation of cytoprotective gene expression programs [66]. Once in the nucleus, NRF2 binds to antioxidant‐response elements (AREs) in the promoters of a wide range of anti‐oxidant genes [67, 68, 69, 70]. These targets include *HMOX1*, *GPX4*, *SLC7A11*, *PRDX6*, ferritin light and heavy chain (*FTL*, *FTH1*), and regulatory subunits of glutamate‐cysteine ligase (*GCLC*, *GCLM*) [71, 72]. Through these downstream effectors, the KEAP1‐NRF2 axis regulates ferroptosis, primarily by enhancing antioxidant defenses and limiting lipid peroxidation [69, 73, 74, 75, 76, 77, 78]. Among NRF2 target genes, *HMOX1* encodes a heme‐degrading enzyme that plays an important role in redox regulation [6, 79]. HMOX1 facilitates the breakdown of heme into biliverdin (subsequently converted to bilirubin, an antioxidant), carbon monoxide, and iron to produce a range of biologically active products including antioxidant, anti‐inflammatory, and cytoprotective compounds. However, HMOX1 exhibits context‐dependent complex roles in regulating ferroptosis: inhibition versus promotion [80, 81, 82, 83, 84, 85, 86, 87, 88, 89]. The exact causes of these pleiotropic functions of HMOX1 are unclear, although it is presumably regulated through iron and ROS balance (ROS‐scavenging effects of heme metabolites vs ROS‐stimulated effects by iron). In general, a proper increase of the cellular HMOX1 is frequently considered detoxifying, cytoprotective, and supportive of cell survival.

Growing evidence suggests that ferroptosis inhibition offers promising therapeutic potential for a range of diseases [90, 91]. Consequently, there is an increasing need for identifying and developing novel ferroptosis inhibitors and druggable protein targets. Inhibitors of ferroptosis are classified into several groups, including iron chelators, endogenous radical‐trapping antioxidants (RTAs), synthetic RTAs, deuterated polyunsaturated fatty acids, lipoxygenase inhibitors, and GPX4 activators [91, 92]. In ferroptosis research, considerable effort is devoted to assessing the reducing capacity of candidate ferroptosis inhibitor compounds [91, 93, 94, 95, 96, 97, 98]. In contrast, their potential to induce ROS is rarely evaluated, despite the potential relevance of ROS‐generating compounds in modulating redox‐adaptive responses.

In this study, we explore the previously unrecognized role of ERM proteins in ferroptosis regulation. Beyond their canonical function as membrane‐actin linkers, we reveal that ERM proteins modulate ferroptotic sensitivity through redox‐adaptive signaling. ERM inhibition triggers a modest actin‐dependent ROS surge that activates the KEAP1‐NRF2 pathway and induces expression of antioxidant genes, including *HMOX1*, ultimately conferring ferroptosis resistance. These findings are supported by ex vivo and in vivo models of ferroptosis‐associated tissue injury, in which ERM inhibition mitigates damage. Furthermore, unrelated ROS‐inducing compounds similarly activate NRF2‐HMOX1 and suppress ferroptosis, suggesting a broader group of ferroptosis inhibitors that operate through ROS‐NRF2‐HMOX1 mediated redox‐adaptive mechanisms [99].

## 2 Results

### 2.1 ERM Inhibition Attenuates Ferroptosis

**FIGURE 1:**
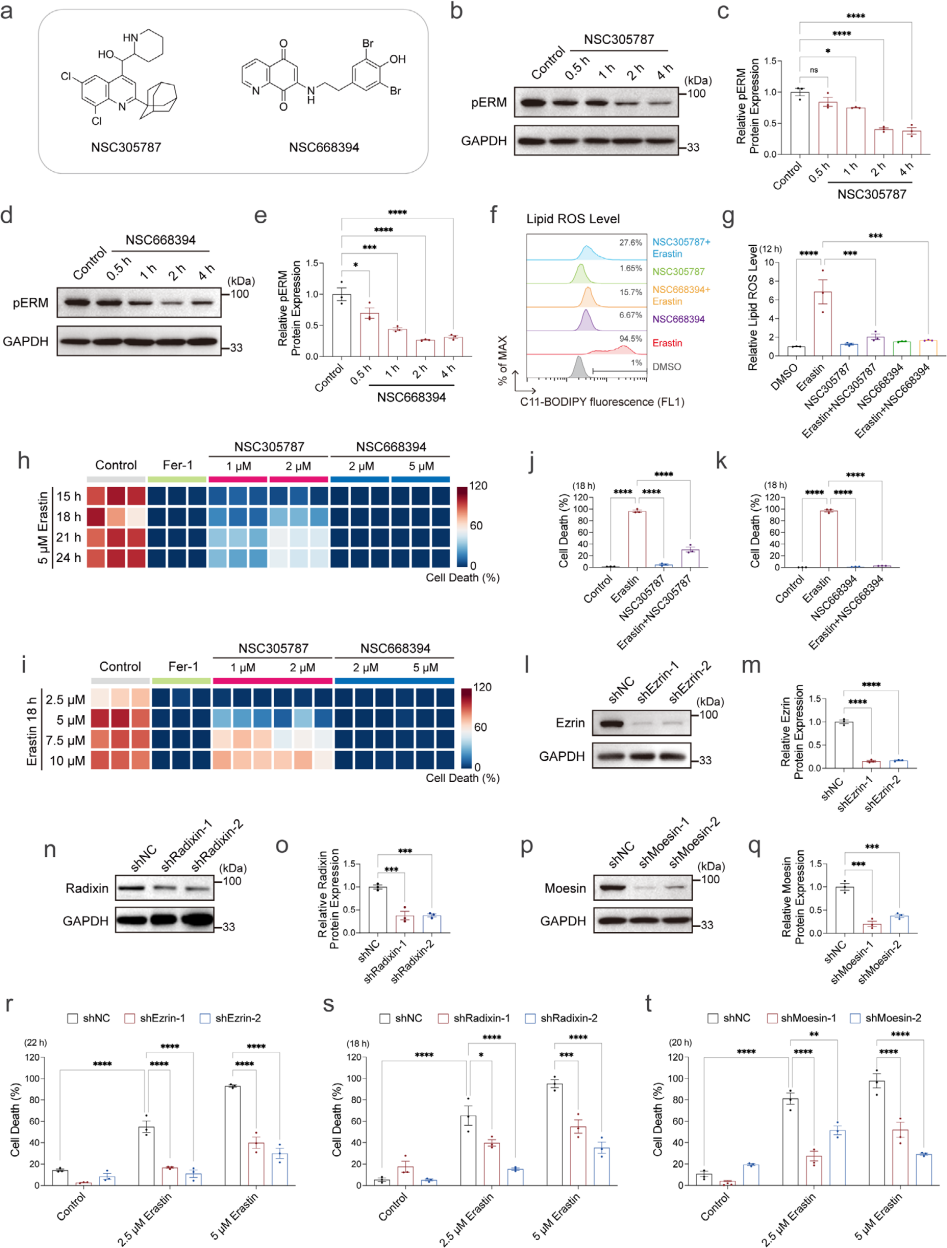
The ERM inhibition attenuates erastin‐induced ferroptosis. (a) Chemical structures of ERM inhibitors NSC305787 and NSC668394. (b,c) Western blot analysis and quantification of pERM protein levels in HT‐1080 cells with NSC305787 (2 µm) treatment at indicated times. (d,e) Western blot analysis and quantification of pERM protein levels in HT‐1080 cells with NSC668394 (5 µm) treatment at indicated times. (f,g) Lipid peroxidation levels measured by C11‐BODIPY fluorescence in HT‐1080 cells treated with DMSO, Erastin (5 µm), NSC305787 (2 µm), or NSC668394 (5 µm), either individually or in combination with Erastin, for 12 h. (h) Cell death measurement of HT‐1080 cells treated with Erastin (5 µm) in combination with Fer‐1 (2 µm), NSC305787 (1 and 2 µm, pre‐treated 3 h), or NSC668394 (2 and 5 µm) for 15–24 h. Dead cells were labeled with Propidium iodide. (i) Cell death measurement of HT‐1080 cells treated with increasing concentrations of Erastin (2.5, 5, 7.5, and 10 µm) in combination with Fer‐1 (2 µm), NSC305787 (1 and 2 µm, pre‐treated 3 h), or NSC668394 (2 and 5 µm) for 18 h. Dead cells were labeled with Propidium iodide. (j) Cell death measurement of HT‐1080 cells treated with DMSO, Erastin (5 µm) and NSC305787 (2 µm) for 18 h. Dead cells were labeled with Propidium iodide. (k) Cell death measurement of HT‐1080 cells treated with DMSO, Erastin (5 µm) and NSC668394 (5 µm) for 18 h. Dead cells were labeled with Propidium iodide. (l,m) Western blot analysis and quantification of Ezrin protein levels in shNC (non‐targeting control) and Ezrin knockdown cells. (n,o) Western blot analysis and quantification of Radixin protein levels in shNC and Radixin knockdown cells. (p,q) Western blot analysis and quantification of Moesin protein levels in shNC and Moesin knockdown cells. (r) Cell death measurement of shNC and shEzrin HT‐1080 cells treated with Erastin (2.5 and 5 µm) for 22 h. Dead cells were labeled with Propidium iodide. (s) Cell death measurement of shNC and shRadixin HT‐1080 cells treated with Erastin (2.5 and 5 µm) for 18 h. Dead cells were labeled with Propidium iodide. (t) Cell death measurement of shNC and shMoesin HT‐1080 cells treated with Erastin (2.5 and 5 µm) for 20 h. Dead cells were labeled with Propidium iodide. Data and error bars are mean ± SEM, *n* = 3 biologically independent experiments in c, e, g, j–k, m, o and q–t. *p < 0.05; **p < 0.01; ***p < 0.001; ****p < 0.0001; n.s., not significant. All *p* values were calculated using a one‐way or two‐way analysis of variance (ANOVA).

To identify novel ferroptosis‐modulating compounds, we utilized HT‐1080 fibrosarcoma cells, a widely used model in ferroptosis research. An exploratory screen of cytoskeleton‐targeting compounds revealed two ERM inhibitors, NSC305787 and NSC668394 (Figure 1a), which markedly attenuated ferroptotic cell death at relatively low concentrations compared with other compounds in the screen (Figure S1a–h). These two compounds are previously reported to suppress ERM protein activity by inhibiting their phosphorylation‐dependent activation at conserved threonine residues (T567 in Ezrin, T564 in Radixin, T558 in Moesin) [31, 48, 51]. Western blot analysis confirmed that both inhibitors reduced phosphorylated ERM (pERM) levels [99, 100, 101, 102, 103], using an antibody recognizing the phosphorylated conserved epitope GRDKYKpTLRQIR (Figure 1b–e). Consistent with their established bioactivity, both compounds inhibited cell proliferation at the experimental concentrations, whereas higher doses under prolonged treatment induced noticeable cytotoxicity (Figure S2a–j). Treatment with either ERM inhibitor significantly attenuated erastin‐induced ferroptotic cell death, as evidenced by decreased Propidium iodide/Hoechst staining and reduced lipid peroxidation (Figure 1f–k; Figure S3a–g). This protective effect was similarly observed under cystine deprivation, which, like erastin, induces ferroptosis by limiting intracellular cystine (Figure S4a,d). In contrast, protection was not consistently observed in ferroptosis triggered by the GPX4 inhibitors RSL3 or ML210 (Figure S4b,c,e,f), likely reflecting mechanistic differences between erastin‐induced and GPX4 inhibition‐induced ferroptosis [104]. Based on this specificity, we focused subsequent analyses on erastin‐induced ferroptosis. Notably, NSC305787 and NSC668394 exhibit limited antioxidant capacity in vitro compared to Vitamin C or Ferrostatin‐1 (Fer‐1) (Figure S5a,b). Moreover, their protective effect appeared selective for ferroptosis, as neither compound conferred protection against other forms of regulated cell death, including apoptosis or cuproptosis (Figure S5c–f).

Despite their high structural similarity (Figure S6a), ERM family members displayed distinct expression levels at both the mRNA and protein levels in HT‐1080 cells (Figure S6b–g). To define their contribution to ferroptosis and provide genetic validation for the inhibitor‐based observations, each ERM gene was individually silenced (Figure 1l–q; Figure S7a–c,i–n), or wild‐type Ezrin, Radixin, or Moesin was overexpressed (Figure S8a–l). Knockdown of any single ERM member conferred a clear, although partial, protective effect against erastin‐induced ferroptosis (Figure 1r–t; Figure S7d–h), whereas overexpression of any ERM protein sensitized cells to ferroptotic cell death (Figure S8m–r). Together, these data identify NSC305787 and NSC668394 as previously unrecognized ferroptosis‐suppressing compounds and establish ERM proteins as new modulators of ferroptosis.

### 2.2 Phosphorylation of the Actin‐Binding Domain of ERM Proteins Regulates Ferroptosis

**FIGURE 2:**
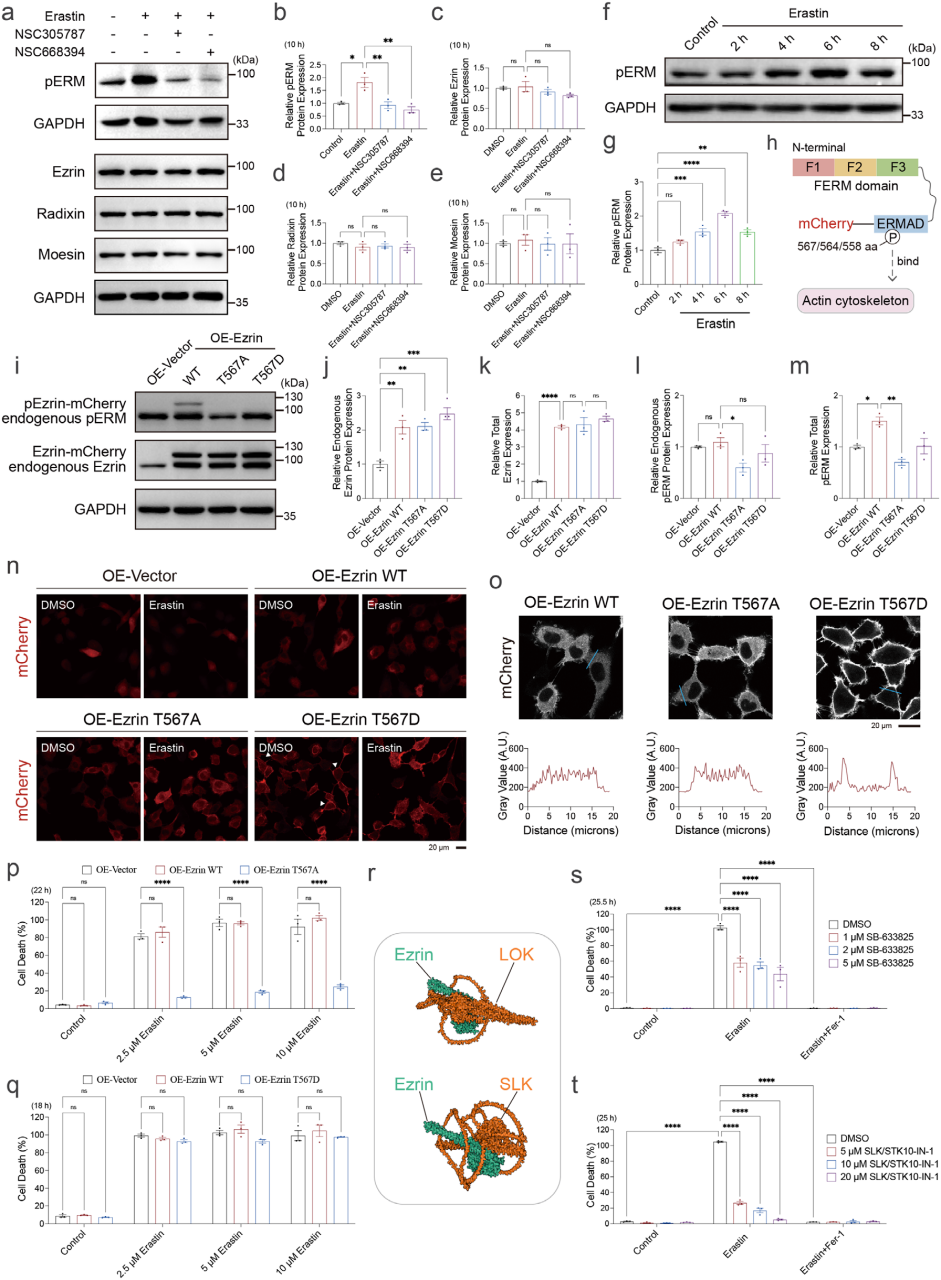
Phosphorylation of ERM proteins within the actin‐binding domain regulates erastin‐induced ferroptosis. (a) Western blot analysis of pERM, Ezrin, Radixin, and Moesin protein levels in HT‐1080 cells treated with the indicated combination of Erastin (5 µm), NSC305787 (2 µm), and NSC668394 (5 µm) for 10 h. (b–e) Quantification of pERM (b), Ezrin (c), Radixin (d), and Moesin (e) protein levels in HT‐1080 cells from a. (f,g) Western blot analysis and quantification of pERM protein levels in HT‐1080 cells treated with Erastin (5 µm) for 2–8 h. (h) Schematic illustration of ERM protein domain structure and site of Thr567/564/558, with mCherry fused to the C terminus as used in the overexpression constructs. F1–F3: subdomain/modules of the FERM domain. (i–m) Western blot analysis and quantification of endogenous Ezrin (j), total Ezrin (endogenous Ezrin and Ezrin‐mCherry) (k), endogenous pERM (l), and total pERM (endogenous pERM and mCherry‐tagged pERM) (m) protein levels in HT‐1080 cells overexpressing vector control, Ezrin WT, Ezrin T567A, or Ezrin T567D. All constructs are mCherry‐tagged. The upper and middle panels in i were detected using anti‐pERM and anti‐Ezrin antibodies, respectively. (n,o) Representative confocal SUM images (n) and single‐plane views (o) of HT‐1080 cells overexpressing vector control, Ezrin WT, T567A, or T567D, treated with DMSO or Erastin (5 µm) for 12 h. White arrowheads highlight membrane‐localized Ezrin T567D‐mCherry signals in overexpressing cells. Fluorescence intensity along the blue line is plotted below. All constructs are mCherry‐tagged. Images were acquired using a confocal microscopy with a 40x objective, capturing data through mCherry channels. Confocal imaging was repeated twice, with each including 25 cells. (p) Cell death measurement of HT‐1080 cells overexpressing vector control, Ezrin WT, or Ezrin T567A treated with increasing concentrations Erastin (2.5, 5, and 10 µm) for 22 h. Dead cells were labeled with SYTOX‐Green. (q) Cell death measurement of HT‐1080 cells overexpressing vector control, WT, or Ezrin T567D treated with increasing concentrations Erastin (2.5, 5, and 10 µm) for 18 h. Dead cells were labeled with SYTOX‐Green. (r) Schematic illustration of the predicted interaction between LOK/SLK and Ezrin based on AlphaFold3 modeling. (s) Cell death measurement of HT‐1080 cells treated with SB‐633825 (1, 2, and 5 µm, pre‐treated 9 h) and Erastin (10 µm) for 25.5 h. Dead cells were labeled with Propidium iodide. (t) Cell death measurement of HT‐1080 cells treated with SLK/STK10‐IN‐1 (5, 10, and 20 µm, pre‐treated 9 h) and Erastin (10 µm) for 25 h. Dead cells were labeled with Propidium iodide. Data and error bars are mean ± SEM, *n* = 3 biologically independent experiments in b–e, g, j–m, p, q and s, t. *p < 0.05; **p < 0.01; ***p < 0.001; ****p < 0.0001; n.s., not significant. All *p* values were calculated using a one‐way or two‐way analysis of variance (ANOVA).

Molecular docking analysis predicted that NSC305787 and NSC668394 may interact with the C‐terminal actin‐binding domain of ERM proteins and interfere with their phosphorylation (Figure S9a) [48]. Notably, total ERM protein levels remained largely unchanged following treatment with either compound (Figure S9b–g). Individual overexpression of ERM proteins was associated with variable changes in both endogenous and exogenous pERM levels, independent of expression magnitude (Figure 2i–m; Figure S9h–m; Figure S8a–l). To assess the relevance of ERM phosphorylation in erastin‐induced ferroptosis, the expression and phosphorylation status of ERM proteins were examined following erastin treatment alone or in combination with the inhibitors. Erastin treatment markedly increased pERM levels (Figure 2a,b,f,g), whereas co‐treatment with NSC305787 or NSC668394 effectively suppressed this phosphorylation (Figure 2a,b). In contrast, total ERM protein levels remained unchanged both before and after erastin treatment, and were similarly unaffected when comparing erastin treatment alone with erastin in combination with either ERM inhibitor (Figure 2a,c–e). To directly examine the functional relevance of ERM phosphorylation, we generated stable cell lines overexpressing mCherry‐tagged wild‐type Ezrin, a non‐phosphorylatable mutant (T567A, threonine to alanine), or a phosphomimetic mutant (T567D, threonine to aspartic acid) (Figure 2h–k). In contrast to wild‐type Ezrin, cells expressing Ezrin‐T567A exhibited reduced endogenous pERM levels compared to those expressing wild‐type Ezrin (Figure 2i,l,m). Although the T567D mutation impairs recognition by the pERM antibody [105], Ezrin‐T567D expression was confirmed through plasmid sequencing (Figure S9n). Confocal microscopy imaging revealed a pronounced accumulation of Ezrin T567D near the plasma membrane, consistent with its role in linking the cell membrane and the actin cytoskeleton (Figure 2n,o, white arrowheads) [48, 106, 107]. Functionally, overexpression of Ezrin‐T567A attenuated erastin‐induced ferroptosis relative to wild‐type Ezrin, whereas Ezrin‐T567D did not confer protection (Figure 2p,q). Given that ERM proteins are phosphorylated by upstream kinases such as LOK and SLK (Figure 2r), which are well‐established regulators of ERM activation at sites like Ezrin T567, we next tested whether inhibition of these kinases modulates ferroptosis [108, 109]. Treatment with their respective inhibitors, SB‐633825 (LOK inhibitor) and SLK/STK10‐IN‐1 (SLK inhibitor), conferred protection against ferroptosis (Figure 2s,t; Figure S9o,p), consistent with the effects of direct ERM inhibition. Collectively, these findings highlight the regulatory role of ERM phosphorylation at the C‐terminal actin‐binding domain in controlling ferroptotic sensitivity.

### 2.3 Actin Cytoskeleton Mediates Ferroptosis and ERM‐Dependent Regulation

**FIGURE 3:**
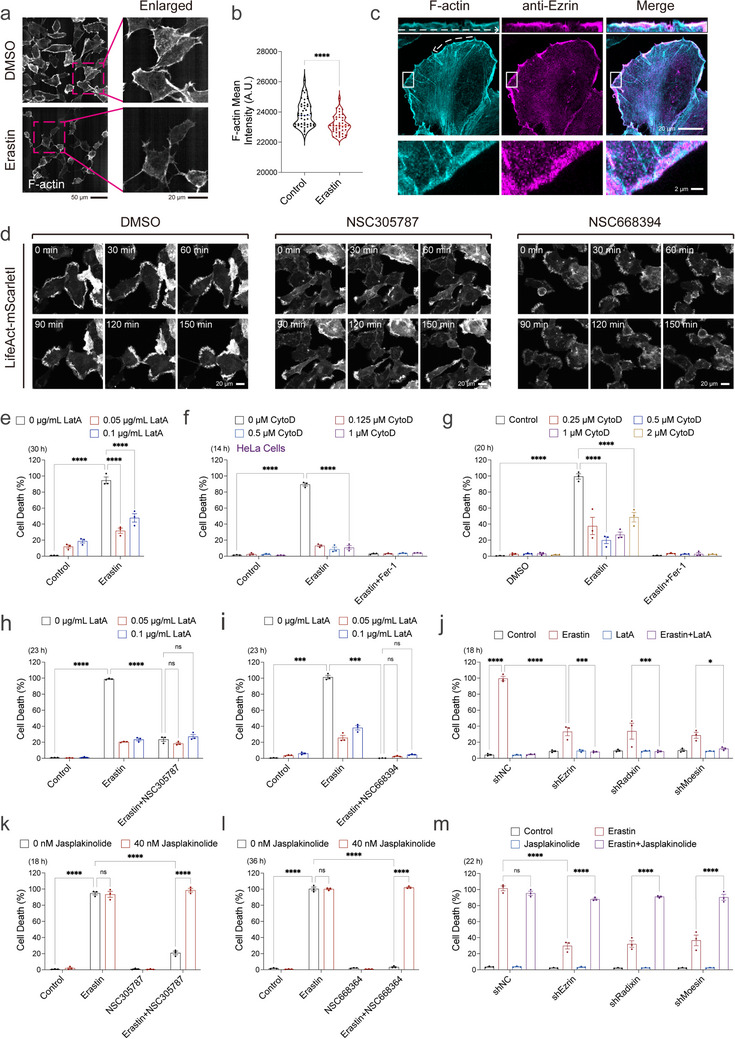
ERM‐Actin axis regulates erastin‐induced ferroptosis. (a) Representative confocal microscopy images of phalloidin‐stained HT‐1080 cells treated with DMSO or erastin (5 µm) for 10 h. Images were acquired as SUM projections using a confocal microscopy with a 60x objective, capturing data through the FITC channel. The image on the right shows an enlarged view of the area within the red box. Confocal imaging was repeated twice (*n* > 30). (b) Quantification of fluorescence intensity in HT‐1080 cells from a (*n* = 46). (c) Representative confocal microscopy images of HT‐1080 cells stained with phalloidin (F‐actin) and anti‐Ezrin antibody. Upper graphs display straightened lines along the cell periphery (white dashed arrow), and lower panels show magnified views of regions marked by white rectangles. Images were acquired as MAX projections using a 60x objective. Confocal imaging was repeated twice (*n* > 30). (d) Time‐lapse imaging of LifeAct‐mScarletI‐expressed HT‐1080 cells treated with DMSO, NSC305787 (2 µm), or NSC668394 (5 µm). Confocal imaging was performed twice (*n* > 30). The time point marked as 0 min represents the start of the movie, with a ∼15–30 min gap between chemical treatment and the movie start. (e) Cell death measurement of HT‐1080 cells treated with LatA (0.05 and 0.1 µg/mL) and Erastin (5 µm) for 30 h. Dead cells were labeled with Propidium iodide. (f) Cell death measurement of HeLa cells treated with CytoD (0.125, 0.5, and 1 µm) and Erastin (10 µm) for 14 h. Dead cells were labeled with Propidium iodide. (g) Cell death measurement of HT‐1080 cells treated with CytoD (0.25, 0.5, 1, and 2 µm) and Erastin (5 µm) for 20 h. Dead cells were labeled with Propidium iodide. (h) Cell death measurement of HT‐1080 cells treated with the indicated combination of Erastin (5 µm), NSC305787 (2 µm), and LatA (0.05 and 0.1 µg/mL) for 23 h. Dead cells were labeled with Propidium iodide. (i) Cell death measurement of HT‐1080 cells treated with the indicated combination of Erastin (5 µm), NSC668394 (5 µm), and LatA (0.05 and 0.1 µg/mL) for 23 h. Dead cells were labeled with Propidium iodide. (j) Cell death measurement of shERM HT‐1080 cells treated with the indicated combination of Erastin (5 µm) and LatA (0.05 µg/mL) for 18 h. Dead cells were labeled with Propidium iodide. (k) Cell death measurement of HT‐1080 cells treated with the indicated combination of Erastin (5 µm), NSC305787 (2 µm), and jasplakinolide (40 nm) for 18 h. Dead cells were labeled with Propidium iodide. (l) Cell death measurement of HT‐1080 cells treated with the indicated combination of Erastin (5 µm), NSC668394 (5 µm), and jasplakinolide (40 nm) for 36 h. Dead cells were labeled with Propidium iodide. (m) Cell death measurement of shERM HT‐1080 cells treated with the indicated combination of Erastin (5 µm) and jasplakinolide (40 nm) for 22 h. Dead cells were labeled with Propidium iodide. Data and error bars are mean ± SEM, *n* = 3 biologically independent experiments in e–m. *p < 0.05; **p < 0.01; ***p < 0.001; ****p < 0.0001; n.s., not significant. All *p* values were calculated using a one‐way or two‐way analysis of variance (ANOVA).

To further investigate the mechanism through which ERM proteins regulate ferroptosis, RNA sequencing was conducted following ERM inhibitor treatment or during ferroptosis progression. Our transcriptomic analysis indicated that ERM inhibition alters the expression of genes associated with ferroptosis, actin cytoskeleton organization, and cellular responses to oxidative stress (Figures S10a–g and S11a–f, and Table S1, Supporting information). However, the statistical significance of enrichment did not reach a stringent threshold, which may reflect limitations in sample size and the timing of sample collection. To further investigate the role of the actin cytoskeleton, we stained ferroptotic cells with phalloidin and observed a decrease in F‐actin signals during ferroptosis (Figure 3a,b). ERM inhibitor treatment also altered F‐actin organization at the cell periphery (Figure S11g), in line with the observed colocalization of endogenous Ezrin and F‐actin in this region (Figure 3c). Live‐cell imaging using LifeAct‐mScarletI further showed that ERM inhibition led to a modest early reduction in F‐actin levels (Figure 3d) [56, 110]. To further verify whether perturbing the actin cytoskeleton alone affects ferroptosis, we treated cells with the actin polymerization inhibitors Latrunculin A (LatA; F‐actin depolymerization confirmed in Figure S11h), Cytochalasin D (CytoD), as well as the Arp2/3 complex inhibitor CK‐666. All three compounds consistently attenuated erastin‐induced ferroptosis (Figure 3e–g; Figure S12a–i). Consistently, silencing of inverted formin 2 (INF2), a key regulator of F‐actin assembly, also attenuated ferroptosis [111, 112, 113, 114] (Figure S13a–l). To directly test whether actin remodeling mediates the protective effects of ERM inhibition, ERM‐inhibited cells were co‐treated with LatA or with the F‐actin stabilizer jasplakinolide [115]. Disrupting actin polymerization with LatA did not further enhance the ferroptosis protection conferred by ERM inhibitor treatment or ERM knockdown (Figure 3h–j). In contrast, stabilizing F‐actin with jasplakinolide markedly reversed the protection afforded by ERM inhibition (Figure 3k–m). Together, these results demonstrate that the actin cytoskeleton is a critical effector of ferroptotic cell death and that ERM proteins regulate ferroptosis through their control of actin organization.

### 2.4 ERM Inhibition Regulates Ferroptosis through Actin‐Mediated ROS Elevation

**FIGURE 4:**
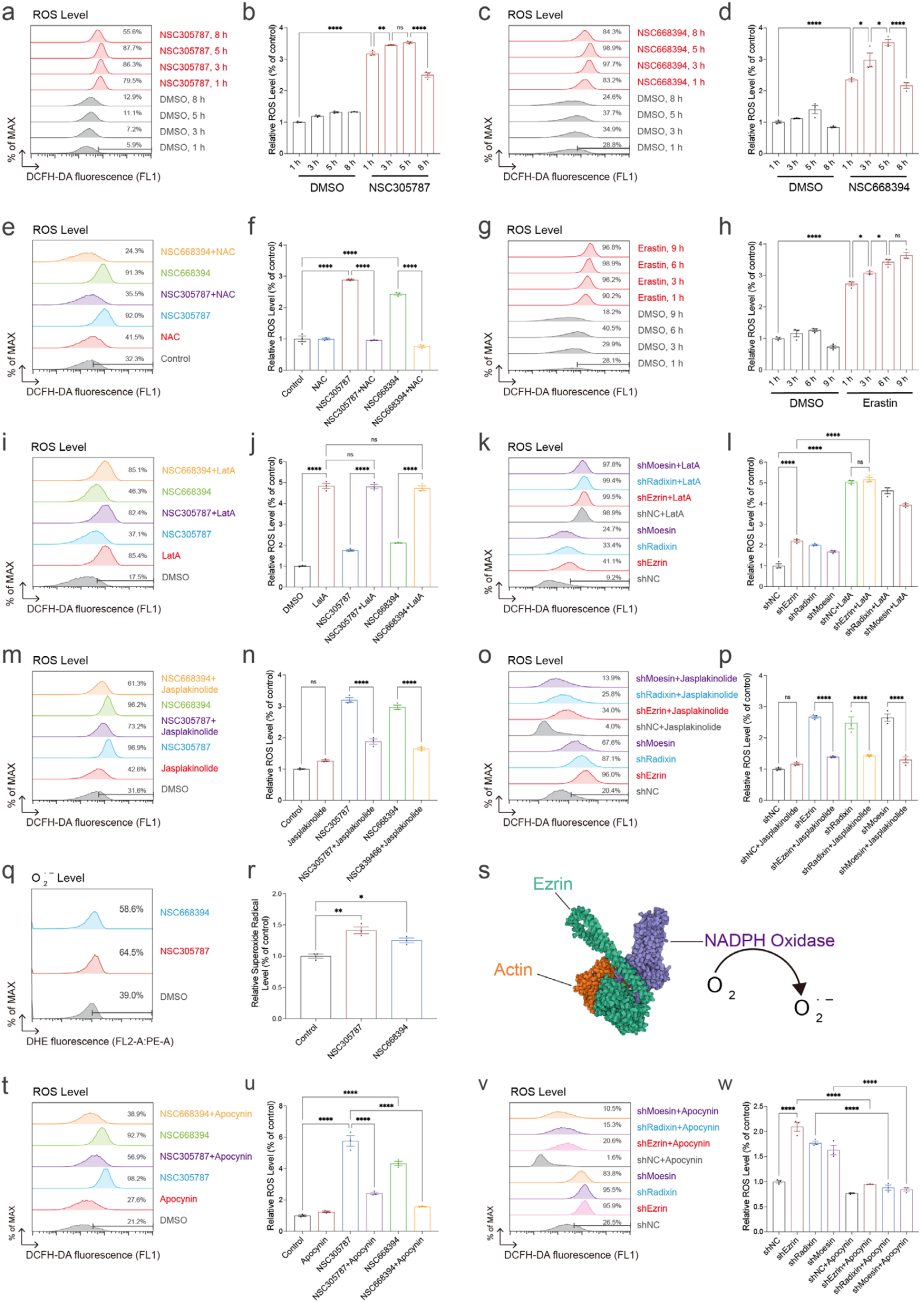
ERM inhibition induces actin‐dependent ROS elevation. (a) ROS levels measured by DCFH‐DA staining in HT‐1080 cells treated with DMSO, NSC305787 (2 µm) over the indicated time course. (b) Quantification of ROS levels from a. (c) ROS levels measured by DCFH‐DA staining in HT‐1080 cells treated with DMSO, NSC668394 (5 µm) over the indicated time course. (d) Quantification of ROS levels from c. (e) ROS levels measured by DCFH‐DA staining in HT‐1080 subjected to individual or combined treatments with NSC305787 (2 µm), NSC668394 (5 µm), or NAC (500 µm) for 3 h. (f) Quantification of ROS levels from e. (g) ROS levels measured by DCFH‐DA staining in HT‐1080 cells treated with DMSO, Erastin (5 µm) over the indicated time course. (h) Quantification of ROS levels from g. (i) ROS levels measured by DCFH‐DA staining in HT‐1080 cells pre‐treated with LatA (0.1 µg/mL) for 2 h following by co‐treatment with LatA (0.1 µg/mL) and either NSC305787 (2 µm) or NSC668394 (5 µm) for an additional 2 h. (j) Quantification of ROS levels from i. (k) ROS levels measured by DCFH‐DA staining in shEzrin‐2, shRadixin‐1, shMoesin‐2 HT‐1080 cells treated with LatA (0.05 µg/mL) for 3 h. (l) Quantification of ROS levels from k. (m) ROS levels measured by DCFH‐DA staining in HT‐1080 cells pre‐treated with jasplakinolide (50 nm) for 2 h followed by co‐treatment with jasplakinolide (50 nm) and either NSC305787 (2 µm) or NSC668394 (5 µm) for an additional 2 h. (n) Quantification of ROS levels from m. (o) ROS levels measured by DCFH‐DA staining in shEzrin‐2, shRadixin‐1, shMoesin‐2 HT‐1080 cells treated with jasplakinolide (50 nm) for 4 h. (p) Quantification of ROS levels from o. (q) Superoxide anion levels measured by dihydroethidium (DHE) staining in HT‐1080 cells treated with DMSO, NSC305787 (2 µm), or NSC668394 (5 µm) for 4 h. (r) Quantification of ROS levels from q. (s) Schematic showing AlphaFold3‐predicted structures of Ezrin, actin, and NOX2. (t) ROS levels measured by DCFH‐DA staining in HT‐1080 cells pre‐treated with Apocynin (20 µm) for 2 h followed by co‐treatment with Apocynin (20 µm) and either NSC305787 (2 µm) or NSC668394 (5 µm) for an additional 2 h. (u) Quantification of ROS levels from t. (v) ROS levels measured by DCFH‐DA staining in shEzrin‐2, shRadixin‐1, shMoesin‐2 HT‐1080 cells treated with Apocynin (20 µm) for 4 h. (w) Quantification of ROS levels from v. Data and error bars are mean ± SEM, *n* = 3 biologically independent experiments in b, d, f, h, j, l, n, p, r, u, and w. *p < 0.05; **p < 0.01; ***p < 0.001; ****p < 0.0001; n.s., not significant. All *p* values were calculated using a one‐way or two‐way analysis of variance (ANOVA).

The aforementioned RNA sequencing data suggested a potential link between ERM inhibition and redox regulation. To explore this, we measured intracellular ROS levels in HT‐1080 cells following treatment with the ERM inhibitors NSC305787 or NSC668394. Both compounds induced a rapid surge in ROS levels shortly after treatment (Figure 4a–d), which was effectively reduced by the ROS scavenger N‐acetylcysteine (NAC) (Figure 4e,f). However, this increase was transient, with ROS levels declining at later time points, indicating a brief oxidative burst rather than sustained ROS production. Notably, the magnitude of ROS elevation induced by ERM inhibitors was less sustained than that induced by the ferroptosis inducer erastin (Figure 4g,h). Given the established interaction between ERM proteins and the actin cytoskeleton, and the known role of actin dynamics in redox regulation, we next explored whether ERM inhibitor‐induced ROS production is actin‐dependent [116, 117]. Both ERM dephosphorylation and LatA treatment elevated ROS levels, however, their combination did not produce a further increase compared to each treatment alone (Figure 4i–l). This observation is consistent with the possibility that they induce ROS through a shared or converging mechanism. Intriguingly, co‐treatment with the F‐actin stabilizer jasplakinolide and ERM dephosphorylation markedly blunted the ROS surge, suggesting that stabilized F‐actin interferes with ERM‐mediated ROS signaling (Figure 4m–p). ERM proteins were reported to interact with NOX2 [118], a key NADPH oxidase generating O_2_·^−^ (Figure 4s). Using a superoxide anion (O_2_·^−^) detection kit and flow cytometry, we observed an increase in O_2_·^−^ levels upon ERM inhibitor treatment (Figure 4q,r), consistent with the overall ROS elevation. Co‐treatment with the NOX inhibitor Apocynin reduced the ROS elevation induced by ERM inhibition (Figure 4t–w), indicating that NOX‐mediated superoxide is likely the major ROS species produced. Collectively, these findings support a model in which ERM inhibition disrupts actin organization, thereby triggering transient ROS elevation through an actin‐dependent mechanism.

### 2.5 ERM‐Actin Axis Regulates Ferroptosis via the ROS‐Sensitive KEAP1‐NRF2 Pathway

**FIGURE 5:**
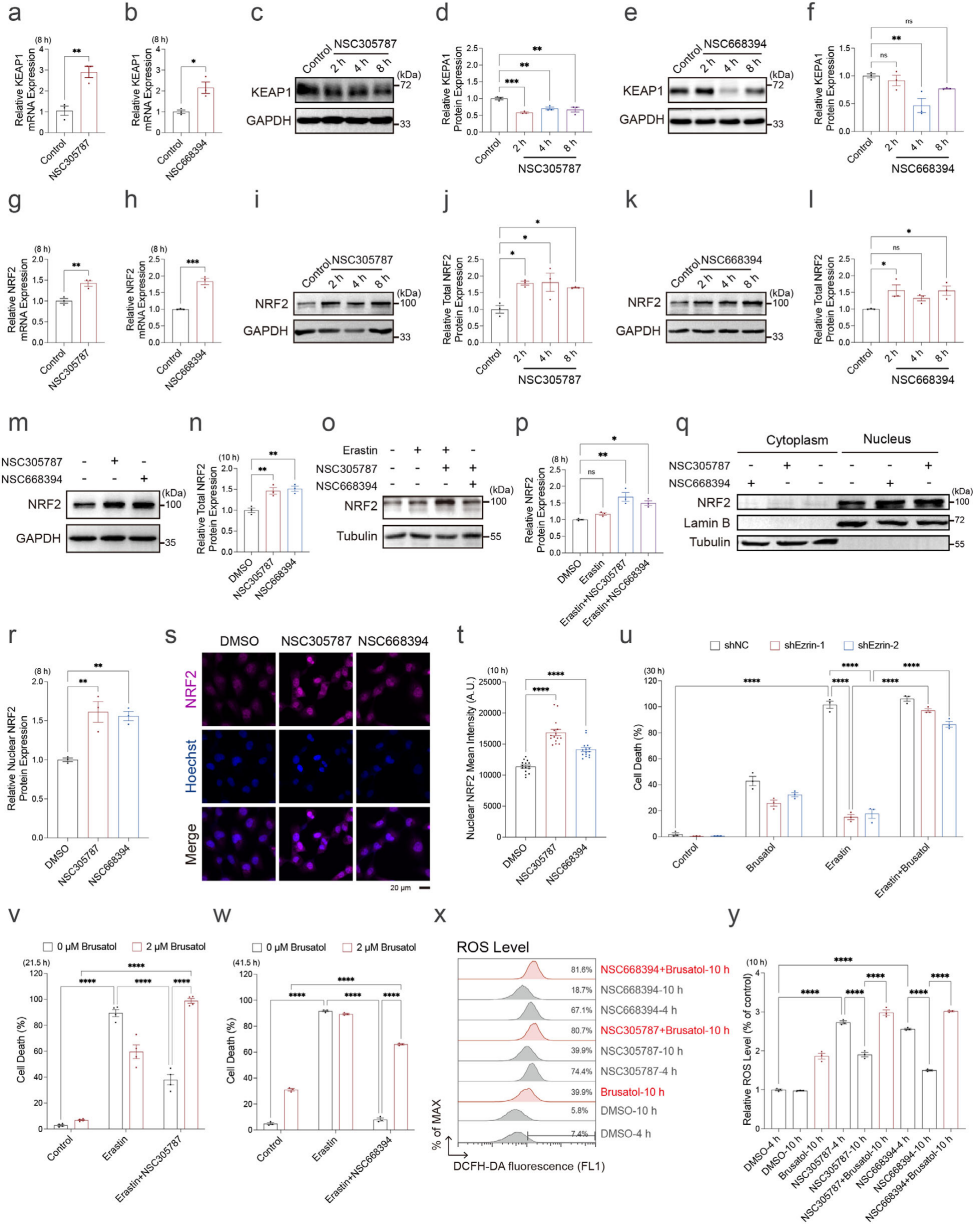
The ERM‐Actin axis regulates erastin‐induced ferroptosis through NRF2. (a) qPCR analysis of *KEAP1* mRNA levels in HT‐1080 cells treated with NSC305787 (2 µm) for 8 h. (b) qPCR analysis of *KEAP1* mRNA levels in HT‐1080 cells treated with NSC668394 (5 µm) for 8 h. (c,d) Western blot analysis and quantification of reduced KEAP1 protein levels in HT‐1080 cells treated with NSC305787 (2 µm) for 2–8 h. (e,f) Western blot analysis and quantification of reduced KEAP1 protein levels in HT‐1080 cells treated with NSC668394 (5 µm) for 2–8 h. (g) qPCR analysis of *NRF2* mRNA levels in HT‐1080 cells treated with NSC305787 (2 µm) for 8 h. (h) qPCR analysis of *NRF2* mRNA levels in HT‐1080 cells treated with NSC668394 (5 µm) for 8 h. (i,j) Western blot analysis and quantification of total NRF2 protein levels in HT‐1080 cells treated with NSC305787 (2 µm) for 2–8 h. (k,l) Western blot analysis and quantification of total NRF2 protein levels in HT‐1080 cells treated with NSC668394 (5 µm) for 2–8 h. (m,n) Western blot analysis and quantification of NRF2 protein levels in HT‐1080 cells treated with DMSO, NSC305787 (2 µm), or NSC668394 (5 µm) for 10 h. (o,p) Western blot analysis and quantification of total NRF2 protein levels in HT‐1080 cells treated with Erastin (5 µm) alone, or in combination with NSC305787 (2 µm) or NSC668394 (5 µm) for 8 h. (q,r) Western blot analysis and quantification of NRF2 protein levels in the cytoplasm and nucleus of HT‐1080 cells treated with DMSO, NSC305787 (2 µm), or NSC668394 (5 µm) for 8 h. (s) Immunofluorescence staining of NRF2 and Hoechst in HT‐1080 cells treated with DMSO, NSC305787 (2 µm), or NSC668394 (5 µm) for 10 h. Images were acquired as SUM projections using a confocal microscopy with a 60x objective, capturing data through DAPI and mCherry channels. Confocal imaging was repeated twice, with each including 37–44 cells. (t) Quantification of nuclear NRF2 mean intensity from s (*n* = 15). (u) Cell death measurement of shNC or shEzrin cells treated with the indicated combination of Brusatol (1 µm, pre‐treated 12.5 h) and Erastin (5 µm) for 30 h. Dead cells were labeled with Propidium iodide. (v) Cell death measurement of HT‐1080 cells treated with the indicated combination of Brusatol (2 µm, pre‐treated 10 h), Erastin (5 µm), and NSC305787 (2 µm) for 21.5 h. Dead cells were labeled with Propidium iodide. (w) Cell death measurement of HT‐1080 cells treated with the indicated combination of Brusatol (2 µm, pre‐treated 10 h), Erastin (5 µm), and NSC668394 (5 µm) for 41.5 h. Dead cells were labeled with Propidium iodide. (x) ROS levels measured by DCFH‐DA staining in HT‐1080 cells treated with DMSO, NSC305787 (2 µm), NSC668394 (5 µm), or Brusatol (1 µm) over the indicated time course. (y) Quantification of ROS levels from x. Data and error bars are mean ± SEM, *n* = 3 biologically independent experiments in a, b, d, f–h, j, l, n, p, r, u, v, w, and y. *p < 0.05; **p < 0.01; ***p < 0.001; ****p < 0.0001; n.s., not significant. All *p* values were calculated using a one‐way or two‐way analysis of variance (ANOVA).

The KEAP1‐NRF2 pathway is a well‐established ROS‐responsive signaling cascade that maintains redox homeostasis and confers cytoprotection under oxidative stress [119]. Given that ERM inhibition elevates ROS levels, we hypothesized that ERM inhibition may attenuate ferroptosis through KEAP1‐NRF2 signaling [120, 121, 122, 123, 124]. qPCR analysis revealed increased *KEAP1* mRNA levels following treatment with ERM inhibitors NSC305787 and NSC668394 (Figure 5a,b). To assess KEAP1 redox status, we examined its electrophoretic mobility by western blot under non‐reducing conditions. ERM inhibition resulted in a reduction of KEAP1 protein at early time points (Figure 5c–f), consistent with its ROS‐induced oxidative modification. To evaluate whether ERM inhibition activates the NRF2 pathway, we measured NRF2 expression and subcellular localization. Treatment with ERM inhibitors elevated both total *NRF2* mRNA and protein levels (Figure 5g–p). Confocal microscopy and biochemical fractionation analyses demonstrated enhanced nuclear translocation of NRF2 upon ERM inhibition (Figure 5q–t). To assess the functional significance of NRF2 in ERM‐mediated ferroptosis regulation, we co‐treated ferroptotic cells with ERM inhibition and NRF2 inhibitors (Brusatol or ML385). While ERM inhibition conferred resistance to ferroptosis, this protection was reversed by NRF2 inhibition, confirming NRF2 as a critical mediator in this process (Figure 5u–w; Figure S14g–j). Accordingly, NRF2 also facilitated the decline in ROS levels following the transient elevation induced by ERM inhibition (Figure 5x,y). Notably, NRF2 inhibition had limited impact on the protective effects of canonical ferroptosis inhibitors such as Fer‐1, liproxstatin‐1 (Lip‐1), or deferoxamine (DFO), suggesting that these inhibitors may operate largely independently of NRF2 signaling, which differs from the ERM inhibitors (Figure S14a–f). To further investigate the role of the actin cytoskeleton in NRF2 subcellular localization, we performed immunofluorescence and nuclear‐cytoplasmic fractionation assays. Treatment with the actin‐depolymerizing agent LatA promoted NRF2 nuclear translocation (Figure S15a,b,e,f), whereas the actin‐stabilizer jasplakinolide reduced NRF2 nuclear translocation (Figure S15c,d,g,h). Functionally, F‐actin disruption mimicked the protective effect of ERM inhibition, which was abolished by Brusatol co‐treatment (Figure S14k–o). In summary, these findings suggest that the ERM‐actin axis regulates ferroptosis through the ROS‐sensitive KEAP1‐NRF2 pathway.

### 2.6 HMOX1 Upregulation Mediates the Anti‐Ferroptotic Effect of ERM Inhibition

**FIGURE 6:**
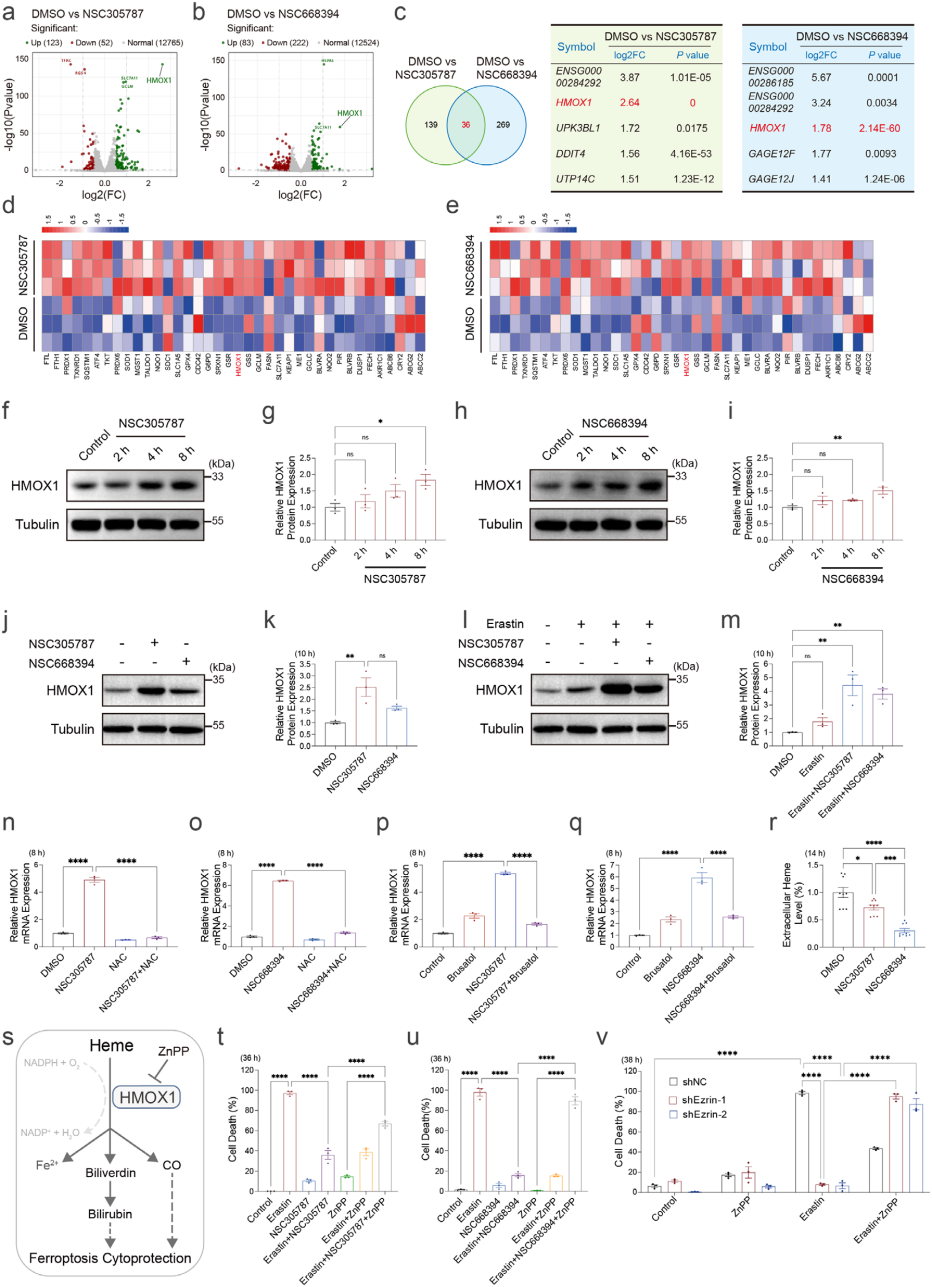
ERM‐Actin‐ROS‐NRF2 regulates ferroptosis through upregulating HMOX1. (a) Volcano plot from RNA sequencing analysis showing *HMOX1* upregulation in HT‐1080 cells treated with NSC305787 (2 µm) (*n* = 3). (b) Volcano plot from RNA sequencing analysis showing *HMOX1* upregulation in HT‐1080 cells treated with NSC668394 (5 µm) (*n* = 3). (c) Venn diagram depicting overlapping genes among two comparisons: DMSO vs NSC305787, and DMSO vs NSC668394. The two tables display the top 5 differentially expressed genes based on their log2 fold change values (*n* = 3). (d) Heatmap of NRF2 target gene expression comparing DMSO and NSC305787‐treated cells. The heatmap colors represent gene expression levels transformed as log10 (FPKM + 0.000001) (*n* = 3). (e) Heatmap of NRF2 target gene expression comparing DMSO and NSC668394‐treated cells. The heatmap colors represent gene expression levels transformed as log10 (FPKM + 0.000001) (*n* = 3). (f,g) Western blot analysis and quantification of HMOX1 protein levels in HT‐1080 cells treated with NSC305787 (2 µm) for 2–8 h. (h,i) Western blot analysis and quantification of HMOX1 protein levels in HT‐1080 cells treated with NSC668394 (5 µm) for 2–8 h. (j,k) Western blot analysis and quantification of HMOX1 protein levels in HT‐1080 cells treated with DMSO, NSC305787 (2 µm), or NSC668394 (5 µm) for 10 h. (l,m) Western blot analysis and quantification of HMOX1 protein levels in HT‐1080 cells treated with the indicated combination of Erastin (5 µm), NSC305787 (2 µm), or NSC668394 (5 µm) for 10 h. (n) qPCR analysis of HMOX1 mRNA levels in HT‐1080 cells after 8 h treatment with DMSO, NSC305787 (2 µm), NAC (500 µm) or their combination. (o) qPCR analysis of HMOX1 mRNA levels in HT‐1080 cells after 8 h treatment with DMSO, NSC668394 (5 µm), NAC (500 µm) or their combination. (p) qPCR analysis of HMOX1 mRNA levels in HT‐1080 cells after 8 h treatment with DMSO, NSC305787 (2 µm), Brusatol (1 µm) or their combination. (q) qPCR analysis of HMOX1 mRNA levels in HT‐1080 cells after 8 h treatment with DMSO, NSC668394 (5 µm), Brusatol (1 µm) or their combination. (r) Measurement of extracellular heme levels in HT‐1080 cells treated with DMSO, NSC305787 (2 µm), or NSC668394 (5 µm) for 14 h (*n* = 9). (s) Schematic illustrating heme catabolism pathway mediated by HMOX1. (t) Cell death measurement of HT‐1080 cells treated with the indicated combination of ZnPP (10 µm, pre‐treated 3 h), Erastin (5 µm), and NSC305787 (2 µm) for 36 h. Dead cells were labeled with Propidium iodide. (u) Cell death measurement of HT‐1080 cells treated with the indicated combination of ZnPP (10 µm, pre‐treated 5.5 h), Erastin (5 µm), and NSC668394 (5 µm) for 36 h. Dead cells were labeled with Propidium iodide. (v) Cell death measurement of shNC or shEzrin cells treated with the indicated combination of ZnPP (10 µm, pre‐treated 5 h) and Erastin (5 µm) for 38 h. Dead cells were labeled with Propidium iodide. Data and error bars are mean ± SEM, *n* = 3 biologically independent experiments in g, i, k, m–q and t–v. *p < 0.05; **p < 0.01; ***p < 0.001; ****p < 0.0001; n.s., not significant. All *p* values were calculated using a one‐way or two‐way analysis of variance (ANOVA).

To elucidate downstream effectors of NRF2 signaling induced by ERM inhibition, we examined genes showing significant fold changes in RNA sequencing data from ERM inhibitor‐treated HT‐1080 cells. Hundreds of genes were differentially expressed (Table S1). Among these, *HMOX1*, a key antioxidant gene transcriptionally activated by NRF2, showed the most significant and consistent upregulation in ERM inhibitor‐treated cells compared to DMSO controls [52] (Figure 6a–e). This finding was validated by qPCR, which confirmed robust *HMOX1* induction following treatment with either NSC305787 or NSC668394 (Figure S16a–g). Western blot analysis further demonstrated elevated HMOX1 protein levels upon ERM inhibitor treatment alone (Figure 6f–k) and in combination with erastin (Figure 6l,m). To determine whether HMOX1 induction by ERM inhibition is ROS‐NRF2‐dependent, we found that its upregulation was suppressed by both the ROS scavenger NAC and the NRF2 inhibitor Brusatol in cells co‐treated with ERM inhibitors (Figure 6n–q), indicating that ERM inhibition‐induced HMOX1 expression relies on the ROS‐NRF2 axis. Supporting this, extracellular heme levels, the metabolic substrate essential for HMOX1 protein function [125], were altered upon ERM inhibition (Figure 6r,s). To assess whether HMOX1 is functionally required for the ferroptosis resistance conferred by ERM inhibition, we used Zinc Protoporphyrin IX (ZnPP), a competitive heme analog that inhibits HMOX1 activity. ZnPP reversed the protective effects of both ERM inhibitors and Ezrin knockdown on ferroptosis, despite its intrinsic radical‐scavenging activity in vitro (Figure 6t–v; Figure S16h), indicating that HMOX1 activity is essential for this resistance. In contrast, ZnPP had minimal effect on the protection conferred by canonical ferroptosis inhibitors such as Fer‐1, Lip‐1, or DFO, highlighting the specificity of the ERM‐HMOX1 axis (Figure S16i–k). Furthermore, ferroptosis resistance induced by actin cytoskeleton perturbation (via LatA, CK‐666, or INF2 knockdown) was also reversed by ZnPP (Figure S16l–n), further supporting the role of HMOX1 as a downstream effector of the ERM‐actin‐ROS‐NRF2 signaling. Together, these results suggest that ERM inhibition attenuates ferroptosis by inducing *HMOX1*, thereby enhancing redox buffering and promoting ferroptosis resistance.

### 2.7 ERM Inhibition Suppresses Ferroptosis‐Relevant Tissue Damage Ex Vivo and In Vivo

**FIGURE 7:**
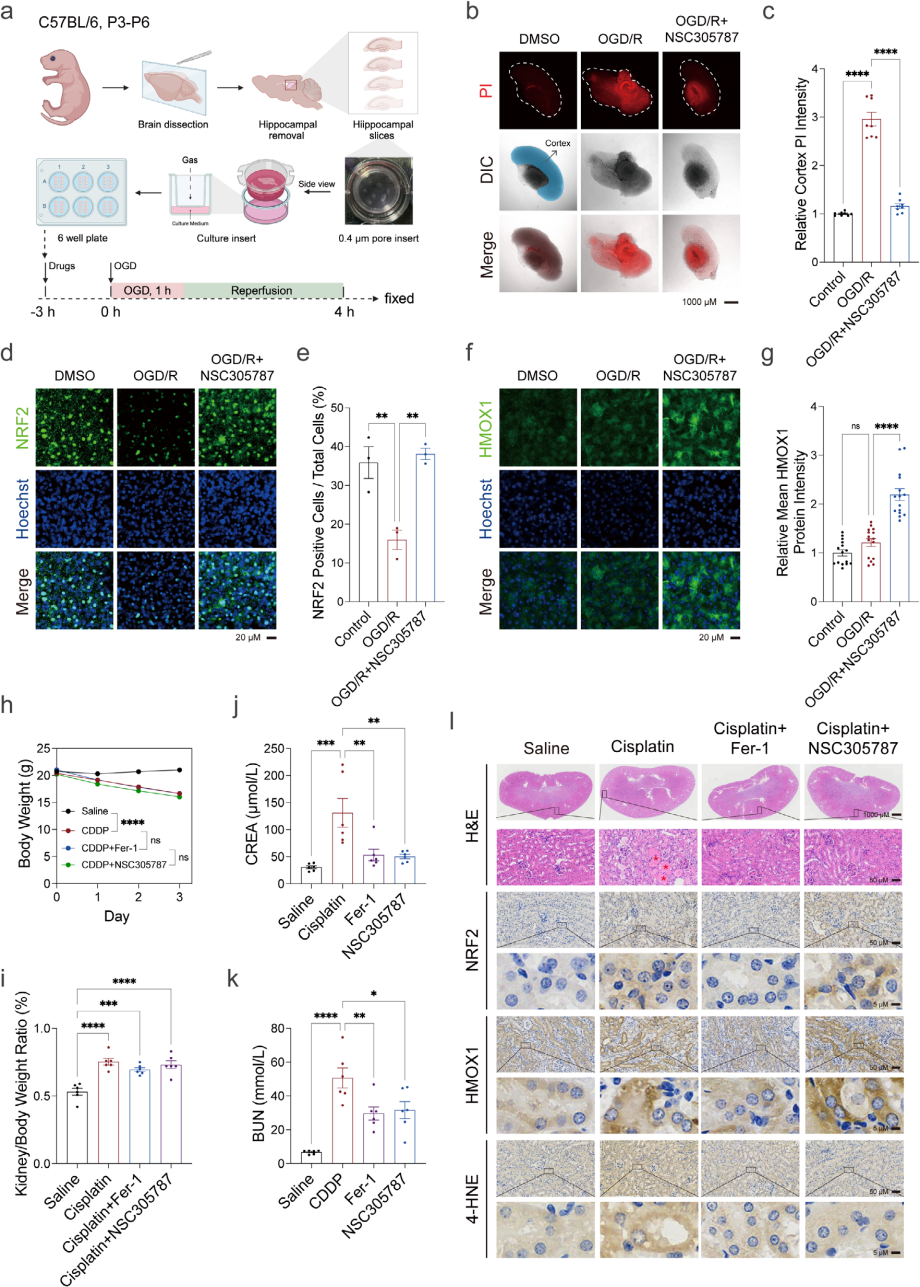
ERM serves as a therapeutic target in ex vivo and in vivo models. (a) Experimental protocol for establishing the OGD/R brain slice model in C57BL/6 neonatal mice from postnatal days 3 to 6. (b) PI staining of OEHSC treated with NSC305787 (5 µm) for 4 h. Images were acquired as single Z‐planes projections using a confocal microscopy with a 2x objective, capturing data through mCherry channels (*n* = 8). (c) PI intensity quantified from b (*n* = 8). (d) Immunofluorescence staining of NRF2 (green) and DAPI (blue) in OGD/R OEHSC treated with NSC305787 (5 µm) for 4 h. Images were acquired as SUM projections using a confocal microscopy with a 20x objective, capturing data through DAPI and FITC channels. (e) Quantification of fraction of NRF2 positive cells from d (*n* = 3). NRF2 positive cell refers to cells with bright NRF2 nucleus staining. (f) Immunofluorescence staining of HMOX1 (green) and DAPI (blue) in OGD/R OEHSC treated with NSC305787 (5 µm) for 4 h, observed using confocal microscopy to assess HMOX1 levels. Images were acquired as SUM projections using a confocal microscopy with a 20x objective, capturing data through DAPI and FITC channels. (g) Quantification of mean HMOX1 intensity from f (*n* = 15 areas). (h) Body weight changes over three days in saline control, cisplatin‐treated, and treatment groups receiving Fer‐1 (5 mg/kg) or NSC305787 (2.5 mg/kg) (*n* = 6). (i) Kidney/Body weight ratio in saline control, cisplatin‐treated, and treatment groups receiving Fer‐1 (5 mg/kg) or (2.5 mg/kg) NSC305787 (*n* = 6). (j) Creatinine (CREA) levels in saline control, cisplatin‐treated, and treatment groups receiving (5 mg/kg Fer‐1) or NSC305787 (2.5 mg/kg) (*n* = 6). (k) Blood urea nitrogen (BUN) levels in saline control, cisplatin‐treated, and treatment groups receiving 5 mg/kg Fer‐1 or 2.5 mg/kg NSC305787 (*n* = 6). (l) Representative H&E staining and immunochemistry staining of mice kidney slices with NRF2, HMOX1, and 4‐HNE antibodies. The enlarged view in the lower panel corresponds to the region within the black box in the upper panel for each staining group. Red asterisks indicate large eosinophilic casts during acute kidney injury. Data and error bars are mean ± SEM, *n* = 6 biologically independent experiments in h–k. *p < 0.05; **p < 0.01; ***p < 0.001; ****p < 0.0001; n.s., not significant. All *p* values were calculated using a one‐way or two‐way analysis of variance (ANOVA).

An unexpected finding was that short‐term pretreatment with the ERM inhibitors NSC305787 or NSC668394, followed by compound withdrawal, revealed more sustained activation of the NRF2‐HMOX1 signaling axis by NSC305787 compared to NSC668394 (Figure S17a–h). The superior effect of NSC305787 over NSC668394 in this context may result from differences in compound stability, as NSC668394 likely has a shorter half‐life following single‐dose treatment [52]. In vivo, no overt adverse effects were observed after NSC305787 administration (2.5 mg/kg, i.p., single dose), consistent with previous reports using a similar dosing regimen (2.4 mg/kg/day for five days) [48]. Encouraged by these findings, we explored the therapeutic potential of NSC305787 in ex vivo and in vivo models relevant to ferroptosis. We first employed an oxygen‐glucose deprivation/reperfusion (OGD/R) model using organotypic entorhinal‐hippocampal slice cultures (OEHSC) [1, 126, 127] (Figure 7a). Propidium iodide (PI) staining showed that ERM inhibition by NSC305787 markedly reduced cell death in the cortex regions of OGD/R brain slices (Figure 7b,c). Immunofluorescence staining showed that NRF2 expression decreased in the injury model but was maintained after NSC305787 treatment (Figure 7d,e), and HMOX1 protein levels were increased in the treated brain slices (Figure 7f,g). To evaluate the in vivo relevance, we employed a cisplatin‐induced acute kidney injury (AKI) model, a severe condition known to involve ferroptosis [128, 129, 130, 131, 132, 133, 134]. Cisplatin administration produced marked body weight loss, increased kidney‐to‐body weight ratio, and elevated serum markers of kidney injury including blood urea nitrogen and creatinine (Figure 7h–k). Although neither NSC305787 nor Fer‐1 fully restored body weight, both compounds appreciably rescued blood urea nitrogen and creatinine levels (Figure 7h–k). Histological analysis by hematoxylin and eosin (H&E) staining revealed that NSC305787 preserved renal architecture and cellular morphology in cisplatin‐treated mice, comparable to the protective effects observed with Fer‐1 (Figure 7l; Figure S17i). Additionally, NSC305787 upregulated NRF2 and HMOX1 protein levels and reduced lipid peroxidation, as evidenced by decreased 4‐HNE staining (Figure 7l; Figure S17i). Collectively, these findings suggest that ERM inhibition suppresses ferroptosis‐relevant tissue injuries both ex vivo and in vivo.

### 2.8 ROS‐Inducing Compounds as a Distinct Group of NRF2‐Dependent Ferroptosis Inhibitors

**FIGURE 8:**
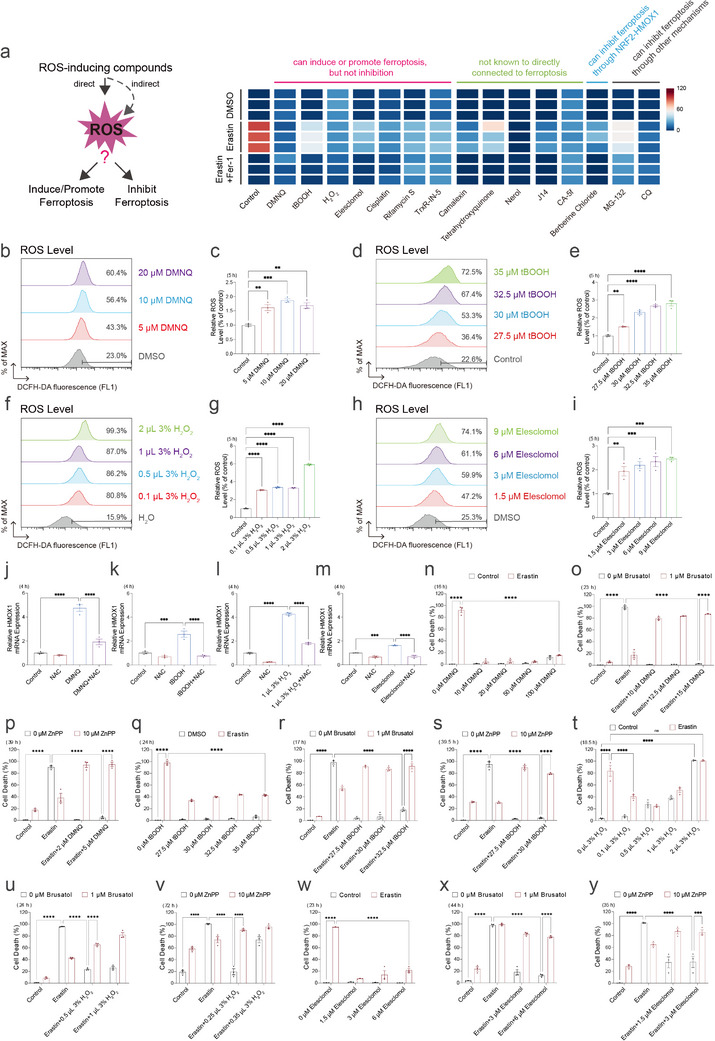
ROS‐inducing compounds attenuates erastin‐induced ferroptosis via NRF2‐HMOX1 signaling. (a) Cell death measurement of HT‐1080 cells treated with the indicated combination of Erastin (5 µm), MG‐132 (0.4 µm), CQ (25 µm), or DMNQ (10 µm), tBOOH (27.5 µm), 0.5 µL 3% H_2_O_2_ per 1.5 mL GM (Growth medium, DMEM high‐glucose medium plus serum), Elesclomol (12 µm), Cisplatin (4 µm), Camalexin (40 µm), Tetrahydroxyquinone (200 µm), Rifamycin S (10 µm), Nerol (140 µm), TrXR‐IN‐5 (0.25 µm), Berberine chloride (1 µm), J14 (10 µm), CA‐5f (12.5 µm), and Fer‐1 (2 µm) at 16 h. Dead cells were labeled with Propidium iodide. The schematic on the left illustrates that ROS can be generated directly or indirectly by these pro‐oxidants, with the subsequent impact on ferroptosis, whether promoting or inhibiting, remaining unclear. (b) ROS levels measured by DCFH‐DA staining in HT‐1080 cells treated with DMSO and increasing concentrations DMNQ (5, 10, and 20 µm) for 5 h. (c) Quantification of ROS levels from b. (d) ROS levels measured by DCFH‐DA staining in HT‐1080 cells treated with H_2_O and increasing concentrations tBOOH (27.5, 30, 32.5, and 35 µm) for 5 h. (e) Quantification of ROS levels from d. (f) ROS levels measured by DCFH‐DA staining in HT‐1080 cells treated with H_2_O and increasing concentrations 3% (W/V) H_2_O_2_ (0.1, 0.5, 1, and 2 µL 3% H_2_O_2_ add to 1.5 mL GM) for 5 h. (g) Quantification of ROS levels from f. (h) ROS levels measured by DCFH‐DA staining in HT‐1080 cells treated with DMSO and increasing concentrations Elesclomol (1.5, 3, 6, and 9 µm) for 5 h. (i) Quantification of ROS levels from h. (j) qPCR analysis of HMOX1 mRNA levels in HT‐1080 cells after 4 h treatment with DMSO, DMNQ (10 µm), NAC (500 µm) or their combination. (k) qPCR analysis of HMOX1 mRNA levels in HT‐1080 cells after 4 h treatment with H_2_O, tBOOH (30 µm), NAC (500 µm) or their combination. (l) qPCR analysis of HMOX1 mRNA levels in HT‐1080 cells after 4 h treatment with H_2_O, 3% H_2_O_2_ (1 µL 3% H_2_O_2_ add to 1.5 mL GM), NAC (500 µm) or their combination. (m) qPCR analysis of HMOX1 mRNA levels in HT‐1080 cells after 4 h treatment with DMSO, Elesclomol (3 µm), NAC (500 µm) or their combination. (n) Cell death measurement of HT‐1080 cells treated with Erastin (10 µm) and increasing concentrations DMNQ (10, 20, 50, and 100 µm) for 16 h. Dead cells were labeled with Propidium iodide. (o) Cell death measurement of HT‐1080 cells treated with the indicated combination of Brusatol (1 µm, pre‐treated 10 h), Erastin (5 µm), and DMNQ (10, 12.5, and 15 µm) for 23 h. Dead cells were labeled with Propidium iodide. (p) Cell death measurement of HT‐1080 cells treated with the indicated combination of ZnPP (10 µm, pre‐treated 4 h), Erastin (10 µm), and DMNQ (2 and 5 µm) for 39 h. Dead cells were labeled with Propidium iodide. (q) Cell death measurement of HT‐1080 cells treated with the indicated concentration of tBOOH (27.5, 30, 32.5, and 35 µm, pre‐treated 4 h) and erastin (10 µm) for 24 h. Dead cells were labeled with Propidium iodide. (r) Cell death measurement of HT‐1080 cells treated with the indicated combination of Brusatol (1 µm, pre‐treated 10 h), tBOOH (27.5, 30, and 32.5 µm, pre‐treated 4 h), and Erastin (5 µm) for 17 h. Dead cells were labeled with Propidium iodide. (s) Cell death measurement of HT‐1080 cells treated with the indicated combination of ZnPP (10 µm, pre‐treated 4 h), tBOOH (27.5 and 30 µm, pre‐treated 4 h), and Erastin (10 µm) for 39.5 h. Dead cells were labeled with Propidium iodide. (t) Cell death measurement of HT‐1080 cells pre‐treated with the indicated amount of 3% (W/V) H_2_O_2_ into 100 µL medium for 5 h. Cell death was measured after adding 10 µm Erastin treatment for 18.5 h. Dead cells were labeled with Propidium iodide. (u) Cell death measurement of HT‐1080 cells pre‐treated with Brusatol (1 µm) for 10 h and 0.5‐1 µL 3% H_2_O_2_ per 1.5 mL GM for 5 h, followed by treatment with Erastin (5 µm), Brusatol (1 µm) and 3% H_2_O_2_ (0.5 and 1 µL 3% H_2_O_2_ per 1.5 mL GM) for 24 h. Dead cells were labeled with Propidium iodide. (v) Cell death measurement of HT‐1080 cells pre‐treated with ZnPP (10 µm) for 5 h and 0.25–0.35 µL 3% H_2_O_2_ per 1.5 mL GM for 9 h, followed by treatment with Erastin (5 µm), ZnPP (10 µm), and 0.25–0.35 µL 3% H_2_O_2_ per 1.5 mL GM for 72 h. Dead cells were labeled with Propidium iodide. (w) Cell death measurement of HT‐1080 cells pre‐treated with the indicated amount of Elesclomol (1.5, 3, and 6 µm) for 4 h. Cell death was measured after adding Erastin (5 µm) treatment for 23 h. Dead cells were labeled with Propidium iodide. (x) Cell death measurement of HT‐1080 cells pre‐treated with Brusatol (1 µm) for 10 h and Elesclomol (3 and 6 µm) for 4 h, followed by treatment with Erastin (5 µm), Brusatol (1 µm) and Elesclomol (3 and 6 µm) for 44 h. Dead cells were labeled with Propidium iodide. (y) Cell death measurement of HT‐1080 cells pre‐treated with ZnPP (10 µm) and Elesclomol (1.5 and 3 µm) for 4 h, followed by treatment with Erastin (5 µm), ZnPP (10 µm), and Elesclomol (1.5 and 3 µm) for 35 h. Dead cells were labeled with Propidium iodide. Data and error bars are mean ± SEM, *n* = 3 biologically independent experiments in c, e, g, i–y. *p < 0.05; **p < 0.01; ***p < 0.001; ****p < 0.0001; n.s., not significant. All *p* values were calculated using a one‐way or two‐way analysis of variance (ANOVA).

Ferroptosis is a regulated form of cell death driven by ROS and lipid peroxidation. As such, ferroptosis inhibitors are typically evaluated based on their radical‐trapping antioxidant activity [91, 92, 135]. Although activation of the NRF2‐HMOX1 pathway is known to regulate ferroptosis, it remains largely unexplored whether a distinct group of ferroptosis inhibitors can induce intracellular ROS and suppress ferroptosis through a shared NRF2‐HMOX1‐dependent mechanism. To explore this, we assembled a panel of compounds reported to increase intracellular ROS via distinct mechanisms [136, 137, 138, 139, 140, 141], including direct ROS generators such as hydrogen peroxide (H_2_O_2_), 2,3‐dimethoxy‐1,4‐naphthoquinone (DMNQ), and tert‐butyl hydroperoxide (tBOOH), which are known to promote ferroptosis but not previously reported as inhibitors, as well as other agents such as Camalexin, Tetrahydroxyquinone, Nerol, J14, CA‐5f, elesclomol, cisplatin, Rifamycin S, and TrxR‐IN‐5, which elevate ROS indirectly or through metal‐based cytotoxicity, and compounds like MG‐132, chloroquine (CQ), and berberine chloride, which are reported to suppress ferroptosis via proteasome inhibition, lysosomal disruption, or NRF2‐HMOX1 activation. Remarkably, when applied at appropriately low concentrations, all these treatments consistently attenuated erastin‐induced ferroptosis (Figure 8a; Figure S18a–l). We further validated DMNQ, tBOOH, H_2_O_2_, and elesclomol and confirmed their ability to elevate intracellular ROS levels (Figure 8b–i). Importantly, these pro‐oxidants elevated the expression of HMOX1 as well as other antioxidant genes (Figure 8j–m; Figure S19a–l). Mechanistic analysis indicated that, similar to ERM inhibitors, these ROS‐inducing compounds suppress ferroptosis through activation of the NRF2‐HMOX1 signaling axis, as co‐treatment with Brusatol or ZnPP reversed their protective effects (Figure 8n–y). Collectively, these findings reveal a paradox in ROS biology, whereby high ROS levels drive ferroptosis, yet moderate ROS elevation can trigger protective antioxidant responses that suppress ferroptosis, highlighting the importance of systematically evaluating ROS‐inducing capacity when characterizing ferroptosis inhibitors.

## 3 Discussion

ERM family proteins are canonically recognized as structural linkers organizing the plasma membrane and actin cytoskeleton to facilitate cell migration and metastasis [31]. However, emerging evidence suggests that their biological roles are more pleiotropic (e.g., organizing membrane receptor distribution, scaffolding signaling complexes, and binding RNA independently of actin [142]) and paradoxical than previously appreciated. High Ezrin expression correlates with poor clinical prognosis in several cancers [41, 47], yet reduced Ezrin levels are also associated with adverse outcomes [143, 144], even within the same cancer type such as ovarian carcinoma [145, 146]. Likewise, the small‐molecule ERM inhibitor NSC305787 effectively limits metastasis but shows limited efficacy against primary tumor growth [147].

Our study extends ERM biology to redox regulation and ferroptosis. We demonstrate that the switch between active (phosphorylated) and inactive (nonphosphorylated) ERM states governs cellular sensitivity to oxidative stress through an actin‐ROS‐NRF2‐HMOX1 signaling axis. Pharmacological ERM inhibitors and F‐actin depolymerizers consistently suppress erastin‐induced ferroptosis across cell lines, supporting a conserved regulatory mechanism. The physiological relevance of this mechanism was confirmed in both organotypic brain slice cultures and an in vivo model of cisplatin‐induced kidney injury, where ERM inhibition mitigated ferroptosis‐associated damage. Notably, the inhibitor NSC305787 showed a favorable safety profile in vivo, consistent with prior reports [48].

The mechanistic link between ERM proteins and ferroptosis may help reconcile the paradoxical effects of ERM inhibition in oncology. Active ERMs enhance motility and metastasis, whereas their inactivation elicits antioxidant responses that reduce ferroptotic vulnerability, potentially impairing immune‐mediated tumor clearance [148]. Thus, therapeutic ERM inhibition may inadvertently promote ferroptosis resistance. To counter this, combining ERM inhibitors with agents that activate alternative cell death pathways could be effective. Consistent with this notion, ERM inhibition enhanced cellular sensitivity to MG‐132‐induced apoptosis and copper‐ionophore‐induced cuproptosis, both reliant on ROS signaling. Whether ERM inhibition similarly modulates NRF2 activity under these conditions remains a compelling question for future investigation.

A central goal in ferroptosis research is to uncover novel molecular regulators and therapeutic targets [6, 8, 149, 150]. An intriguing facet of our findings is that various ROS‐inducing compounds, including NSC305787, NSC668394, LatA, DMNQ, tBOOH, H_2_O_2_, elesclomol, can paradoxically protect cells from ferroptosis through a shared NRF2‐HMOX1‐dependent mechanism. These compounds consistently attenuate erastin‐induced ferroptosis when applied at appropriately low concentrations, but not at high doses, reflecting the concept of hormesis, where low levels of a typically harmful stressor elicit adaptive and protective effects [151, 152]. This dual role of ROS underscores the importance of precise dosage control when using ROS inducers for therapeutic purposes, as sublethal ROS can trigger redox‐adaptive responses that delay ferroptosis. These findings suggest that ROS‐inducing compounds represent a distinct and underappreciated class of ferroptosis inhibitors, highlighting the need to systematically assess their ROS‐generating capacity in addition to their radical‐trapping activity [91].

Intriguingly, although erastin induces a clear rise in ROS (∼3.1‐fold at 3 h, Figure 4g,h), itself does not suppress ferroptosis. A possible explanation is that its activation of the NRF2‐HMOX1 axis remains relatively weak, resulting in only modest increases of total NRF2 (∼1.2‐fold at 8 h, Figure 5o,p) and HMOX1 protein levels (∼1.8‐fold at 10 h, Figure 6l,m). In contrast, ROS inducers such as ERM inhibitors elevate ROS to a similar extent (∼3‐fold at 3 h, Figure 4a–d), yet, when co‐treated with erastin, elicit comparable NRF2 activation (∼1.5‐fold at 8 h, Figure 5o,p) together with a substantially stronger HMOX1 upregulation (∼4‐fold at 10 h, Figure 6l,m). The reason erastin generates robust ROS but only minimal HMOX1 activation remains unclear and merits further investigation. Another important consideration is that erastin's primary mechanism, blocking system Xc^−^, depleting glutathione, and impairing GPX4, directly accelerates lipid peroxidation. This creates a dominant pro‐ferroptotic force that outweighs its modest activation of the NRF2‐HMOX1 axis. Under these conditions, the limited induction of HMOX1, which primarily mitigates upstream ROS, is insufficient to counterbalance the ferroptotic cascade once lipid peroxides accumulate. Although ROS elevation is essential for initiating adaptive antioxidant defenses, the magnitude of HMOX1 activation, rather than ROS levels alone, may ultimately determine whether oxidative stress results in cytoprotective adaptation or ferroptotic cell death. Thus, both the amplitude and the mechanistic context of redox signaling should be considered when interpreting ROS‐driven adaptive suppression of ferroptosis.

The redox‐regulatory landscape triggered by ERM inhibition may extend beyond NRF2 alone. In our organotypic entorhinal‐hippocampal slice experiments, NRF2 levels were markedly reduced in the OGD/R group compared to DMSO‐treated controls (Figure 7d,e). In contrast, slices subjected to OGD/R + NSC305787 maintained NRF2 levels comparable to those of the DMSO group (Figure 7d,e). The decrease in NRF2 following OGD/R is consistent with previous work in microglial OGD/R models [153]. These observation on NRF2 appeared different from our in vitro ferroptosis experiments, where total NRF2 increased after erastin treatment and rose even higher with combined erastin + NSC305787 treatment (Figure 5o,p). The reason for this discrepancy remains unclear. One possible explanation is that the OGD/R condition does not completely recapitulate the molecular features of ferroptosis induced by erastin. The mechanistic distinctions between these stress conditions warrant further investigation. In contrast to NRF2, HMOX1 behavior in the OGD/R brain slice culture parallels our in vitro ferroptosis results. HMOX1 remained low in the control slices but was strongly upregulated by OGD/R + NSC305787 treatment (Figure 7f,g). However, given that NRF2 levels in OGD/R + NSC305787 slices were not substantially higher than in DMSO controls, it is unclear how HMOX1 expression becomes markedly elevated under these conditions despite comparable amounts of the transcriptional activator NRF2. One explanation is that transcriptional activation of NRF2 target genes such as HMOX1 does not rely solely on NRF2 abundance but requires coordinated interactions with small MAF proteins, transcriptional co‐activators, and the release of repression mediated by BACH1. Notably, BACH1 is itself regulated by intracellular ROS levels [154, 155]. Elevated ROS promote the nuclear export and subsequent degradation of BACH1, thereby relieving its repression of NRF2‐responsive genes. NSC305787 generates ROS as a pro‐oxidant, potentially compensating for the relatively ROS‐poor OGD environment and enabling more effective HMOX1 transcription despite modest NRF2 levels. Thus, while NRF2 nuclear accumulation is necessary, the effective activation of its downstream genes may depend on the broader redox‐driven transcriptional context, including co‐factor dynamics and the ROS‐mediated regulation of repressors such as BACH1.

Notably, NSC305787 exhibits a sustained cytoprotective effect in vitro even after the compound is washed out (Figure S17a–d). This persistence implies that the ferroptosis‐inhibitory effect may extend beyond the period of direct compound exposure, an aspect that warrants systematic investigation in future studies. It is also noteworthy that although NSC305787 attenuates ferroptosis triggered by erastin or cystine deprivation (Figure 1h–j; Figure S4a), it does not protect against RSL3‐ or ML210‐induced ferroptosis (Figure S4b,c). This divergence likely reflects the distinct mechanisms of these inducers when combined with NSC305787: erastin inhibits system Xc^−^, resulting in glutathione depletion and an indirect loss of GPX4 activity, whereas RSL3 directly and covalently inactivates GPX4 [104]. Furthermore, although both NSC305787 and NSC668394 reduce cell proliferation (Figure S2a–d), NSC668394 showed minimal cytotoxicity (as assessed by plasma membrane rupture using PI staining; Figure S2h–j) within the 24–48 h experimental window used in our ferroptosis assays, whereas NSC305787 caused partial cell death only at higher concentrations (4–8 µm; Figure S2e–g). Importantly, NSC305787 and NSC668394, consistent with the effects of F‐actin polymerization inhibitors, attenuated erastin‐induced ferroptosis across multiple cell lines (Figures S3b–g and S12c–f), supporting that this protective response is general rather than cell‐specific.

Further complexity arises from compounds such as Brusatol. NRF2 inhibition with Brusatol reduced cell death under erastin treatment (Figure 5v; Figure S14a–g,m). Brusatol itself showed no intrinsic ROS‐scavenging activity in the ABTS assay (Figure S16h), yet it has been reported to increase ROS levels [156, 157]. Consistently, our experiments showed that Brusatol treatment alone markedly elevated HMOX1 mRNA expression (Figure 6p). One possible explanation is that Brusatol induces HMOX1 through compensatory transcriptional programs mediated by alternative ROS‐sensitive transcription factors [125]. Because cells in our experiments were pre‐treated with Brusatol prior to erastin exposure, this adaptive HMOX1 upregulation may enhance cellular tolerance to subsequent oxidative stress and thereby attenuate ferroptosis. These findings highlight the complexity of redox‐adaptive signaling and the potential involvement of compensatory transcriptional networks in modulating ferroptotic sensitivity. Moreover, pharmacological inhibition of HMOX1 paradoxically also reduced cell death upon erastin treatment (Figure 6t,u; Figure S16i–n). This effect may arise from the intrinsic antioxidant property of the ZnPP compound (Figure S16h), although similar observations have been reported previously, in which HMOX1 knockdown attenuated ferroptosis [87, 158].

Collectively, our work identifies ERM proteins as modulators of ferroptosis via an actin‐ROS‐NRF2‐HMOX1 signaling cascade and establishes ROS‐inducing compounds as a distinct class of ferroptosis inhibitors. These findings highlight the context‐dependent nature of ROS signaling and the role of compensatory transcriptional networks in shaping ferroptotic sensitivity, providing a conceptual framework for therapeutic strategies in pathological contexts.

## 4 Methods

### 4.1 Cell Lines

The cell lines HT‐1080, 4T1, HeLa, Hep G2, 293T, and 3T3 were obtained from the Shanghai Institute of Cell Biology, Chinese Academy of Sciences, and OVCAR‐8 was sourced from Cellverse Co., Ltd. Prior to experiments, cells were tested for mycoplasma to ensure the absence of contamination. Cells were maintained and subcultured every 1–3 days in cell culture medium, supplemented with 10% fetal bovine serum (FBS, ExCell Bio.) and 1% penicillin/streptomycin (Beyotime). HT‐1080, 293T, Hep G2, HeLa, and 3T3 cells were cultured in Dulbecco's modified Eagle's medium (DMEM, Gibco, #6124229). OVCAR‐8 and 4T1 were cultured in 1640 medium (BasalMedia, #L210KJ). All cultures were kept in a humidified incubator maintained at 37°C with 5% CO_2_.

**Table:** 

Reagent or Resource	Source	Identifier
Antibodies		
Anti‐GAPDH	Proteintech	Cat #60004‐1‐Ig
Anti‐Ezrin	ZenBio, CN	Cat #R24261
Anti‐Radixin	Selleck	Cat #F1012
Anti‐Moesin	Selleck	Cat #A5744;
INF2	Proteintech	Cat #20466‐1‐AP
NRF2	Abcam	Cat #ab31163
HMOX1	ZenBio, CN	Cat #670853
KEAP1 Rabbit mAb	Cell Signaling Technology	Cat #8047
p‐Ezrin/Radixin/Moesin (Thr567/Thr564/Thr558)	ZenBio, CN	Cat #R26293
NRF2 Rabbit mAb	ZenBio, CN	Cat #R380773
Lamin B1 Rabbit mAb	Cell Signaling Technology	Cat #13435
Tubulin	Abmart	Cat #M30109
Goat anti‐Rabbit IgG (H+L) Highly Cross‐Adsorbed Secondary Antibody, Alexa Fluor 594	Invitrogen	Cat #A11037
Anti‐rabbit IgG, HRP‐linked Antibody	Cell Signaling Technology	Cat #7074
Anti‐mouse IgG, HRP‐linked Antibody	Cell Signaling Technology	Cat # 7076
Bacterial strains		
DH5 alpha	TransGen	Cat #CD201‐01
*E. coli* Stbl3	Weidi	Cat #DL1046S
Chemicals, peptides, and recombinant proteins		
Erastin	Sellcek	Cat #S7242
RSL3	Sellcek	Cat #S8155
ML210	MCE	Cat #HY‐100003
NSC305787	MCE	Cat #HY‐18931A; Cat #HY‐18931
NSC668394	MCE	Cat #HY‐115492
Ferrostatin‐1 (Fer‐1)	MCE	Cat #HY‐100579
Jasplakinolide	Santa Cruz	Cat #102396‐24‐7
ML385	TargetMol	Cat #T4360
Zinc Protoporphyrin IX (ZnPP)	MCE	Cat #HY‐101193
Cisplatin	Selleck	Cat #S1166
Cytochalasin D	Glpbio	Cat #GC13440
tert‐butyl hydroperoxide (tBOOH)	MACKLIN	Cat #B802372‐50ml
2,3‐dimethoxy‐1,4‐naphthalenedione (DMNQ)	MCE	Cat #HY‐121026
MG‐132	MCE	Cat #HY‐13259
Z‐VAD	MCE	Cat #HY‐16658B
CuCl_2_	Macklin	Cat #C804816
Elesclomol	Macklin	Cat #E864529
Ammonium tetrathiomolybdate (TTM)	Macklin	Cat #A828261
Liproxstatin‐1(Lip‐1)	Macklin	Cat #950455‐15‐9
Deferoxamine mesylate (DFO)	MCE	Cat #HY‐B0988
SB‐663825	MCE	Cat #HY‐108333

**Table:** 

Reagent or Resource	Source	Identifier
SLK/STK10‐IN‐1	MCE	Cat #HY‐132868
Chloroquine (CQ)	MCE	Cat #HY‐17589A
CK‐666	MCE	Cat #HY‐16926
Latrunculin A (LatA)	Abcam	Cat #ab144290
Brusatol	MCE	Cat #HY‐19543
N‐acetylcysteine (NAC)	Sigma	Cat #A7250
H_2_O_2_	Lircon	Cat #6926378903443
Y‐27632	MCE	Cat #HY‐10071
SMIFH2	MCE	Cat #HY‐16931
NP‐G2‐044	MCE	Cat #HY‐125506
Pfn1‐IN‐1	MCE	Cat #HY‐136808
Benproperine phosphate	MCE	Cat #HY‐114657A
Camalexin	MCE	Cat #HY‐119502
Tetrahydroxyquinone	MCE	Cat #HY‐B1106
Rifamycin S	MCE	Cat #HY‐125365
Nerol	MCE	Cat #HY‐N7063
TrxR‐IN‐5	MCE	Cat #HY‐147803
Berberine chloride	MCE	Cat #HY‐18258
J14	MCE	Cat #HY‐135008
CA‐5f	MCE	Cat #HY‐112698
Apocynin	MCE	Cat #HY‐N0088
C11‐BODIPY	Thermofisher	Cat #D3861
CCK‐8 reagent	Yeasen	Cat #40203ES60
ABTS Free Radical Scavenging Capacity Assay Kit	Solarbio	Cat #BC4775
Reactive oxygen species Assay Kit	Nanjingjiancheng	Cat #E004‐1‐1
Heme Assay Kit	FineTest	Cat #FN240715
Nuclear and Cytoplasmic Protein Extraction Kit	Beyotime	Cat #P0028
Reactive Oxygen Species Assay Kit for Superoxide Anion with DHE	Beyotime	Cat #S0064S
Dulbecco's modified Eagle's medium (DMEM)	Gibco	Cat #6124229
1640 medium	BasalMedia	Cat #L210KJ
Trypsin‐EDTA (0.25%)	Gibco	Cat #25200072
Prestained Protein Ladder (10‐180 kDa)	GeneTech	Cat #R1001‐002
Gold Band Plus 3‐color Regular Range Protein Marker (8‐180 kDa)	Yeasen	Cat #20350ES72
Tween‐20	Beyotime	Cat #ST825
SDS	Biofroxx	Cat #3250
Glycine	Biofroxx	Cat #1275
TBS	Servicebio	Cat #G0001
APS substitute	Beyotime	Cat #ST005
30% Acr‐Bis (29:1)	Beyotime	Cat #ST003
1 M Tris‐HCl, pH = 6.8	Beyotime	Cat #ST768
1.5 M Tris‐HCl, pH = 8.8	Beyotime	Cat #ST768
TEMED	Beyotime	Cat #ST728

**Table:** 

Reagent or Resource	Source	Identifier
PBS	Biosharp	Cat #BL302A
Skimmed milk powder	Shyuanye	Cat #R21306
Skimmed milk powder	Yeasen	Cat #36120ES
FBS	ExCell	Cat #FSP500
Penicillin/streptomycin	Beyotime	Cat #C0222
Trichloromethane	GuangZhou Chemical reagent factory	Cat #GD10
Trizol	Thermofisher	Cat #15596018
Isopropyl alcohol	GuangZhou Chemical reagent factory	Cat #HC16
Ethanol absolute	GuangZhou Chemical reagent factory	Cat #HB15
RIPA buffer	Solarbio	Cat #R0010
PMSF	Solarbio	Cat #P0100
Phosphatase inhibitor	Roche	Cat #4906837001
Phosphatase Inhibitor Cocktail ǁ	Targetmol	Cat #C0003‐1ml
Tris	Biofroxx	Cat #1115GR500
Sample Loading Buffer, 5X	Epizyme	Cat #LT101S
Protein Free Rapid Blocking Buffer	Epizyme	Cat #PS108P
BSA	Epizyme	Cat #PS113
Primary Antibody Dilution Buffer	Beyotime	Cat #P0023A‐100 mL; Cat # P0023D‐100ml
Opti‐MEM	Gibco	Cat ##31985‐070
Polyethylenimine Linear (PEI) MW40000 (rapid lysis)	Yeasen	Cat #40816ES02
Sodium chloride	Macklin	Cat #S805275‐500 g
PEG‐8000	Amresco	Cat #0159‐500G
Polybrene	Glpbio	Cat #GC19206‐1
Puromycin dihydrochloride	MCE	Cat #HY‐B1743A
Hoechst 33342	Beyotime	Cat #C1022
SYTOX‐Green	Keygen	Cat #KGA261
Propidium iodide	Beyotime	Cat #ST511
TritonX‐100	Sigma	Cat #T8787
4% PFA	Servicebio	Cat #G1101
Phalloidin	Yeasen	Cat #40774ES03
Yeast Extract	Sangon Biotech	Cat #A610961‐0500
Ampicillin	Genview	Cat #AA022
Agar	Aladdin	Cat #A501163
Peptone	Sangon Biotech	Cat #A505247‐0500
Critical commercial assays		
Hifair® III first Strand cDNA Synthesis SuperMix for qPCR (gDNA digester plus)	Yeasen	Cat #11141ES60
Hieff® qPCR SYBR Green Master Mix (No Rox)	Yeasen	Cat #11201ES08
Enhanced BCA Protein Assay Kit	Beyotime	Cat #P0009
DNA Gel Extraction Kit	Generay	Cat #GK2043‐200

**Table:** 

Reagent or Resource	Source	Identifier
Seamless Cloning Kit	Beyotime	Cat #D7010S
Mut Express II Fast Mutagenesis Kit V2	Vazyme	Cat #C214‐01
Endo‐Free Plasmid Mini Kit II	Omega	Cat #D6950
jetPRIME siRNA Transfection Reagent	Polyplus	Cat #101000027
Experimental models: Cell lines		
Human: HT‐1080	Shanghai Institute of Cell Biology	RRID: CVCL_0317
Human: OVCAR‐8	Cellverse	RRID: CVCL_1629
Human: Hep G2	Shanghai Institute of Cell Biology	RRID: CVCL_0027
Human: 293T	Shanghai Institute of Cell Biology	RRID: CVCL_0063
Human: HeLa	Shanghai Institute of Cell Biology	RRID: CVCL_0030
Mouse: 4T1	Shanghai Institute of Cell Biology	RRID: CVCL_0125
Mouse: 3T3	Shanghai Institute of Cell Biology	RRID: CVCL_0594
Experimental models: Organisms/strains		
P3‐P6 neonatal mice	Vital River	N/A
6‐7 weeks old male C57BL/6 mice	Zhiyuan	N/A
Oligonucleotides		
Human‐EZRIN‐F: TACCGCGGGCCCGGGATCctcaccagaaaccgaaaatgccga	Tsingke Biotech	N/A
Human‐EZRIN‐R: TGGCGACCGGTGGATCcagggcctcgaactcgtcgat	Tsingke Biotech	N/A
Human‐EZRIN‐T567A‐F: ACAAGTACAAGgCGCTGCGGCAGATCCGGCAG	TIANYI HUIYUAN	N/A
Human‐EZRIN‐T567A‐R: CAGCGcCTTGTACTTGTCCCGGCCTTGCCTCA	TIANYI HUIYUAN	N/A
Human‐EZRIN‐T567D‐F: AGTACAAGgatCTGCGGCAGATCCGGCAGGGC	TIANYI HUIYUAN	N/A
Human‐EZRIN‐T567D‐R: CCGCAGatcCTTGTACTTGTCCCGGCCTTGCC	TIANYI HUIYUAN	N/A
shEzrin‐1: CGTGGGATGCTCAAAGATAAT	Tsingke Biotech	N/A
shEzrin‐2: CCCACGTCTGAGAATCAACAA	Tsingke Biotech	N/A
shMoesin‐1: GCATTGACGAATTTGAGTCTA	Tsingke Biotech	N/A
shMoesin‐2: GCGGATTAACAAGCGGATCTT	Tsingke Biotech	N/A
shRadixin‐1: GCCTTATGTATGGGAAACCAT	Tsingke Biotech	N/A
shRadixin‐2: ATGAGCATGACGACAAGTTAA	Tsingke Biotech	N/A
shINF2‐1: AGCTGCGGAACGAGTTTATCG	Tsingke Biotech	N/A
shINF2‐2: CCGCTTCAGCATTGTCATGAA	Tsingke Biotech	N/A
siNC‐Sense: UUCUCCGAACGUGUCACGUTT	GenePharma	N/A
siNC‐Antisense: ACGUGACACGUUCGGAGAATT	GenePharma	N/A
siINF2‐R: GGAGAUCACUUUCCUCGAUTT	GenePharma	N/A
siINF2‐F: AUCGAGGAAAGUGAUCUCCTT	GenePharma	N/A

**Table:** 

Reagent or Resource	Source	Identifier
Human‐Ezrin‐qPCR‐F: ACCAATCAATGTCCGAGTTACC	YouKang	N/A
Human‐Ezrin‐qPCR‐R: GCCGATAGTCTTTACCACCTGA	YouKang	N/A
Human‐Moesin‐qPCR‐F: GAGGATGTGTCCGAGGAATTG	YouKang	N/A
Human‐Moesin‐qPCR‐R: GTCTCAGGCGGGCAGTAAA	YouKang	N/A
Human‐Radixin‐qPCR‐F: TATGCTGTCCAAGCCAAGTATG	YouKang	N/A
Human‐Radixin‐qPCR‐R: CGCTGGGGTAGGAGTCTATCA	YouKang	N/A
Human‐KEAP1‐qPCR‐F: CTGGAGGATCATACCAAGCAGG	YouKang	N/A
Human‐KEAP1‐qPCR‐R: GGATACCCTCAATGGACACCAC	YouKang	N/A
Human‐NRF2‐qPCR‐F: TCCAGTCAGAAACCAGTGGAT	YouKang	N/A
Human‐NRF2‐qPCR‐R: GAATGTCTGCGCCAAAAGCTG	YouKang	N/A
Human‐SLC7A11‐qPCR‐F: GCGTGGGCATGTCTCTGAC	YouKang	N/A
Human‐SLC7A11‐qPCR‐R: GCTGGTAATGGACCAAAGACTTC	YouKang	N/A
Human‐HMOX1‐qPCR‐F: TTCAAGCAGCTCTACCGCTC	YouKang	N/A
Human‐HMOX1‐qPCR‐R: GGGGGCAGAATCTTGCACT	YouKang	N/A
Human‐GPX4‐qPCR‐F: GAGGCAAGACCGAAGTAAACTAC	YouKang	N/A
Human‐GPX4‐qPCR‐R: CCGAACTGGTTACACGGGAA	YouKang	N/A
Human‐GCLM‐qPCR‐F: TGTCTTGGAATGCACTGTATCTC	Tsingke Biotech	N/A
Human‐GCLM‐qPCR‐R: CCCAGTAAGGCTGTAAATGCTC	Tsingke Biotech	N/A
Human‐PRDX1‐qPCR‐F: CCACGGAGATCATTGCTTTCA	Tsingke Biotech	N/A
Human‐PRDX1‐qPCR‐R: AGGTGTATTGACCCATGCTAGAT	Tsingke Biotech	N/A
Human‐FTH1‐qPCR‐F: CAGCCTGGTCAATTTGTACCT	Tsingke Biotech	N/A
Human‐FTH1‐qPCR‐R: GCCAATTCGCGGAAGAAGTG	Tsingke Biotech	N/A
Human‐GAPDH‐qPCR‐F: GGAGCGAGATCCCTCCAAAAT	YouKang	N/A
Human‐GAPDH‐qPCR‐R: GGCTGTTGTCATACTTCTCATGG	YouKang	N/A
Human‐Actin‐qPCR‐F: CACCATTGGCAATGAGCGGTTC	Tsingke Biotech	N/A
Human‐Actin‐qPCR‐R: AGGTCTTTGCGGATGTCCACGT	Tsingke Biotech	N/A
Recombinant DNA		
Plasmid: pLKO.1‐copGFP‐2A‐puromycin	Tsingke Biotech	N/A
Plasmid: pLKO.1‐MCS‐copGFP‐puromycin	Tsingke Biotech	N/A
Plasmid: pLVX‐MCS‐mCherry‐PGK‐puromycin	QinyunBio	N/A
Plasmid: pLV3‐CMV‐LifeAct‐mScarletI‐Puro	MiaoLing	Cat #P78730
Plasmid: psPAX2	Addgene	N/A
Plasmid: pMDG.2	Addgene	N/A
Software and algorithms		
Prism 9.0	GraphPad	N/A
FlowJo v10	FlowJo, LLC	N/A
Adobe Illustrator	Adobe	N/A
ImageJ	National Institutes of health	N/A
Deposited Data		
Transcriptome sequencing	This paper	SUB14700769 SUB14675389

### 4.2 Chemicals and Reagents

NSC305787 (MCE, #HY‐18931A) (dissolved in DMSO, sonicated for 5 min to make a 10 mm stock solution, aliquoted and stored at −80°C, re‐sonicated for 2 min before use); NSC668394 (MCE, #HY‐115492); Erastin (Sellcek, #S7242) (dissolved in DMSO to make a 10 mm stock solution, aliquoted and stored at −80°C, avoid repeated freeze‐thaw cycles); Jasplakinolide (SANTN CRUZ, #102396‐24‐7); ML385 (TargetMol, #T4360); Zinc Protoporphyrin IX (ZnPP, MCE, #HY‐101193); (1S, 3R)‐RSL3 (Selleck, #S8155); ML210 (MCE, #HY‐100003); Cytochalasin D (CytoD, Glpbio, #GC13440); tert‐butyl hydroperoxide (tBOOH, MACKLIN, #B802372‐50 mL); 2,3‐dimethoxy‐1,4‐naphthalenedione (DMNQ, MCE, #HY‐121026); MG‐132 (MCE, #HY‐13259); Z‐VAD (MCE, #HY‐16658B); CuCl_2_ (Macklin, #C804816); Elesclomol (Macklin, #E864529); Ammonium tetrathiomolybdate (TTM, Macklin, #A828261); SB‐663825 (MCE, HY‐108333); SLK/STK10‐IN‐1 (MCE, #HY‐132868); Chloroquine (CQ, MCE, #HY‐17589A); CK‐666 (MCE, #HY‐16926); Ferrostatin‐1 (Fer‐1, MCE, #HY‐100579); Latrunculin A (LatA, Abcam, #ab144290); Brusatol (MCE, #HY‐19543); Liproxstatin‐1 (Lip‐1, Macklin, #950455‐15‐9); Deferoxamine mesylate (DFO, MCE, #HY‐B0988); 3% H_2_O_2_ (Lircon, #6926378903443); N‐acetylcysteine (NAC, Sigma, #A7250); Cisplatin (CDDP, Selleck, #S1166). Y‐27632 (MCE, #HY‐10071); SMIFH2 (MCE, #HY‐16931); NP‐G2‐044 (MCE, #HY‐125506); Pfn1‐IN‐1 (MCE, #HY‐136808); Benproperine phosphate (MCE, #HY‐114657A); Camalexin (MCE, #HY‐119502); Tetrahydroxyquinone (MCE, #HY‐B1106); Rifamycin S (MCE, #HY‐125365); Nerol (MCE, #HY‐N7063); TrxR‐IN‐5 (MCE, #HY‐147803); Berberine chloride (MCE, #HY‐18258); J14 (MCE, #HY‐135008); CA‐5f (MCE, #HY‐112698); Apocynin (MCE, #HY‐N0088).

### 4.3 Construction of Ezrin Overexpression and Mutant Vectors

Full‐length human EZRIN cDNA was seamlessly cloned (Beyotime, #D7010S) into the pLVX‐MCS‐mCherry‐PGK‐puromycin lentiviral vector (QinyunBio, #QP1699) using the following primers:

Sense Primer: TACCGCGGGCCCGGGATCctcaccagaaaccgaaaatgccga

Anti‐sense Primer: TGGCGACCGGTGGATCcagggcctcgaactcgtcgat

The resulting pLVX‐EZRIN‐mCherry‐PGK‐puromycin lentiviral vector was then subjected to site‐directed mutagenesis (Vazyme, #C214‐01) to generate the EZRIN T567A‐mCherry and T567D‐mCherry lentiviral vectors using the following primers:

(1) $$T567A\left(\mathrm{Thr}\left(\mathrm{ACG}\right)\to \mathrm{Ala}\left(\mathrm{GCG}\right)\right)$$

Sense Primer: ACAAGTACAAGgCGCTGCGGCAGATCCGGCAG

Anti‐sense Primer: CAGCGcCTTGTACTTGTCCCGGCCTTGCCTCA

(2) $$T567D\left(\mathrm{Thr}\left(\mathrm{ACG}\right)\to \mathrm{Asp}\left(\mathrm{GAT}\right)\right)$$

Sense Primer: AGTACAAGgatCTGCGGCAGATCCGGCAGGGC

Anti‐sense Primer: CCGCAGatcCTTGTACTTGTCCCGGCCTTGCC

### 4.4 Lentiviral‐Mediated Gene Overexpression

The pLVX‐EZRIN‐mCherry, pLVX‐EZRIN T567A‐mCherry, pLVX‐EZRIN T567D‐mCherry, pLVX‐Radixin‐mCherry, and pLVX‐Moesin‐mCherry lentiviral expression plasmids were co‐transfected with packaging plasmids psPAX2 and pMD2.G into 293T cells at a molar ratio of 4:3:1. Lentiviral supernatants were harvested at 48 and 72 h post‐transfection, concentrated using PEG‐8000 (Amresco, #0159‐500G). To establish stable cell lines, HT‐1080 cells were infected with the concentrated lentiviral supernatants with 5 µg/mL polybrene (Glpbio, #GC19206‐1) for 24 h, followed by selection with 4 µg/mL puromycin for one week.

### 4.5 shRNA‐Mediated Gene Silencing

shRNA constructs for Ezrin, Moesin, Radixin and INF2 were purchased from Tsingke Biotech Company. shEzrin, shRadixin, and shMoesin were cloned into the pLKO.1‐MCS‐copGFP‐puromycin lentiviral vector, while shINF2 was cloned into the pLKO.1‐copGFP‐2A‐puromycin backbone. The target sequences were as follows:

shEzrin‐1: CGTGGGATGCTCAAAGATAAT

shEzrin‐2: CCCACGTCTGAGAATCAACAA

shMoesin‐1: GCATTGACGAATTTGAGTCTA

shMoesin‐2: GCGGATTAACAAGCGGATCTT

shRadixin‐1: GCCTTATGTATGGGAAACCAT

shRadixin‐2: ATGAGCATGACGACAAGTTAA

shINF2‐1: AGCTGCGGAACGAGTTTATCG

shINF2‐2: CCGCTTCAGCATTGTCATGAA

Lentiviral plasmids containing the above target sequences were co‐transfected with packaging plasmids psPAX2 and pMD2.G into 293T cells at a molar ratio of 4:3:1. Lentiviral supernatants were harvested at 48 and 72 h post‐transfection, concentrated using PEG‐8000 (Amresco, #0159‐500G). To establish stable cell lines, HT‐1080 cells were infected with the concentrated lentiviral supernatants with 5 µg/mL polybrene (Glpbio, #GC19206‐1) for 24 h, followed by selection with 4 µg/mL puromycin for one week.

### 4.6 siRNA‐Mediated Gene Silencing

For siRNA transfection, the siRNA powder was centrifuged at 15 000 rpm for 1 min and dissolved in DEPC water to a concentration of 125 µL per OD_260_. Aliquots of 10 µL were stored at −80°C. HT‐1080 cells were seeded in 6‐well plates at a density of 150 000 cells per well and incubated at 37°C with 5% CO_2_ for 24 h. The transfection mix was prepared by combining 0.2 mL jetPRIME buffer (Polyplus, #101000027) with 5 µL siRNA, followed by the addition of 4 µL jetPRIME reagent. The mixture was gently pipetted to mix thoroughly and then allowed to sit at room temperature. After 15 min, the transfection mix was added to the wells. Cells were incubated for 12 h. Subsequently, the medium was replaced with fresh DMEM for further experiments. Transfected cells were then seeded at a density of 4000 cells per well in a 96‐well plate or 150 000 cells per well in a 6‐well plate and cultured for 24 h to allow for adherence. Cells in the 6‐well plate were collected for Western blotting and qPCR to verify knockdown efficiency. After 22 h of erastin treatment in the 96‐well plate, cell death was analyzed using PI and Hoechst staining.

The target sequences for siINF2 were as follows:

siINF2 Sense primer: GGAGAUCACUUUCCUCGAUTT

siINF2 Anti‐sense primer: AUCGAGGAAAGUGAUCUCCTT

### 4.7 Lentiviral LifeAct‐mScarletI Cell Line Generation

Plasmid pLV3‐CMV‐LifeAct‐mScarletI‐Puro was co‐transfected with psPAX2 and pMD2.G into 293T. Viral supernatants harvested at 24, 48, and 72 h were pooled, filtered (0.45 µm) and used to infect HT‐1080 in one round. Transduced cells were maintained under 2 µg/mL puromycin selection, expanded, and seeded on glass‐bottom dishes with puromycin‐growth medium. After drug treatment, LifeAct‐mScarletI‐labelled actin was imaged by confocal microscopy using 561 nm excitation and 540‐620 nm emission.

### 4.8 Nuclear and Cytoplasmic Protein Extraction

Cytoplasmic and nuclear proteins were extracted with the Beyotime P0027 kit. HT‐1080 cells grown in 10‐cm dishes to 70% confluence were drug‐treated, rinsed with ice‐cold PBS, and scraped.After centrifugation (500 g, 3 min, 4°C), pellets were resuspended in 200 µL ice‐cold buffer A (plus 1 mm PMSF), vortexed 5 s, and kept on ice 15 min. Ten microliters of buffer B were added, vortexed 5 s, incubated 1 min on ice, re‐vortexed 5 s, and centrifuged (16 000 g, 5 min, 4°C); supernatants constituted cytoplasmic proteins. Pellets were washed and extracted with 50 µL nuclear buffer containing 1 mm PMSF, vortexed 15 s every 2 min for 30 min on ice, then centrifuged (16 000 g, 10 min, 4°C) to obtain nuclear proteins. All proteins were quantified by BCA, mixed with loading buffer, denatured at 100°C for 10 min, and then stored at ‐80°C.

### 4.9 Western Blot Analysis

The HT‐1080 cells were seeded in 6‐well plates at a density of 15 × 10^4^ cells per well and cultured for 48 h at 37°C with 5% CO_2_. Following the treatment with the specified agents, the HT‐1080 cells were lysed using RIPA buffer (high) (Solarbio, #R0010) with PMSF (Solarbio, #P0100) and Phosphatase Inhibitor Cocktail II (Targetmol, #C0003‐1 mL). The supernatants were collected for total protein concentration measurements using the BCA Protein Assay Kit (Thermo, #23227). Samples were denatured at 100°C for 10 min using Protein loading buffer (Epizyme, #LT101S). Subsequently, samples were separated by 12% sodium dodecyl sulfate‐polyacrylamide gel electrophoresis (SDS‐PAGE) using molecular weight markers (Western blots used different protein markers depending on availability: 8–180 kDa, Yeasen, #20350ES72, or 10–180 kDa from GeneTech, #R1001‐002), and then transferred onto 0.45 µm polyvinylidene difluoride (PVDF) membranes. The membranes were blocked with 5% skim milk in TBST buffer for 2 h or Protein Free Rapid Blocking Buffer (Epizyme, #PS108P) for 30 min at room temperature, then incubated overnight at 4°C with primary antibodies diluted in dilution solution (Beyotime, #P0023A): GAPDH (at a dilution of 1:20000, Proteintech, #60004‐1‐Ig), Tubulin (at a dilution of 1:2000, Abmart, #M30109), Ezrin (at a dilution of 1:2000, ZenBio, #R24261), Radixin (at a dilution of 1:2000, Selleck, #A5280/#F1012), Moesin (at a dilution of 1:2000, Selleck, #A5744), and p‐Ezrin/Radixin/Moesin (Thr567/Thr564/Thr558) (at a dilution of 1:2000, Antigen sequence: GRDKYKpTLRQIR, p stands for phosphorylation, ZenBio, #R26293), HMOX1 (at a dilution of 1:1000, ZenBio, #670853), KEAP1 (at a dilution of 1:2000, Cell Signaling Technology, #8047), NRF2 (at a dilution of 1:2000, ZenBio, #R380773; Abcam, #ab31163), INF2 (at a dilution of 1:4000, Proteintech, #20466‐1‐AP), Lamin B1 (at a dilution of 1:2000, Cell Signaling Technology, #13435). Corresponding secondary antibodies (at a dilution of 1:5000, CST, #7076S, #7074S) were diluted in TBST and incubated for 3 h at 4°C. Protein bands were visualized using ECL detection reagents (Gel imaging system: Tanon‐5200Multi). ImageJ software was used to analyze and quantify the grayscale of the protein bands, and the data from three independent biological replicates were subjected to statistical analysis.

### 4.10 Immunofluorescence Staining of Cell Cultures

Cells were seeded onto 8‐well chamber slides (Ibidi, #80826) at a density of 5000 cells per well and cultured for 1–2 days before the experiments. Post treatment with the indicated conditions, cells were fixed with 4% paraformaldehyde at 37°C for 15 min, permeabilized with PBS containing 0.5% Triton X‐100 for 10 min, and blocked with 5% BSA for 1 h. Cells were then incubated overnight at 4°C with the indicated primary antibody, followed by incubation with an appropriate fluorescently‐labeled secondary antibody (e.g., diluted 1:2000, Invitrogen, #A11037) at room temperature for 1 h. Hoechst (0.5 µg/mL, Beyotime, #C1022) was used for nuclei staining. Imaging was performed using confocal microscopy.

### 4.11 Phalloidin Staining for F‐Actin

Cells were seeded at a density of 5 × 10^4^ cells per well into 4‐chamber 35 mm glass‐bottomed dishes (tissue culture‐treated, JingAn Bio., China) and cultured for 36 h. Post treatment with the indicated conditions, cells were fixed and stained according to the following steps: residual medium was removed by washing with PBS, followed by fixation with 4% paraformaldehyde at 37°C for 15 min. Next, cells were permeabilized with PBS containing 0.5% Triton X‐100 for 10 min, and then stained with fluorescein isothiocyanate (FITC)‐conjugated phalloidin (1:200, #40735ES75) at 37°C for 15–30 min. After staining, cells were stained with 0.5 µg/mL Hoechst 33342 (Beyotime, #C1022) for nucleus staining. Z stack imaging was done sequentially in FITC and DAPI channels.

### 4.12 Cell Death Assay

Cells were plated in 96‐well plates at a density of 5000 cells per well and incubated overnight at 37°C in a humidified cell culture incubator with 5% CO_2_. After treatments with indicated conditions for a specified period, cells were stained with Hoechst 33342 (1 µg/mL, Beyotime, #C1022), and propidium iodide (PI, #ST511, 5 µg/mL) or SYTOX‐Green (Keygen, #KGA261, 5 µg/mL) to assess total and dead cell counts, respectively. Fluorescence signals were measured using a cell imaging multimode fluorescence plate reader with a 4x objective, capturing data through DAPI and Texas Red or GFP channels. Cell death percentage was calculated by the ratio of PI‐positive or SYTOX Green‐positive cell count to the total Hoechst‐positive cell count. The assay was conducted with three replicate wells per treatment group and was independently repeated three times.

### 4.13 CCK‐8 Cell Viability Assay

Cells were plated in 96‐well plates at a density of 5000 cells per well and incubated overnight at 37°C in a humidified cell culture incubator with 5% CO_2_. After the cells reached 70% confluency, they were treated under the indicated conditions for periods ranging from 0 to 48 h, 10 µL of CCK‐8 reagent (YEASEN, #40203ES60) was added to each well and incubated for 2 h at 37°C in the dark. Absorbance was measured at 450 nm using a microplate reader (SYNERGY4). Cell viability was calculated based on the manufacturer's provided formula. The assay was conducted with three replicate wells per treatment group and was independently repeated three times.

### 4.14 RNA Sequencing

The HT‐1080 cells were seeded in 6‐well plates at a density of 15 × 10^4^ cells per well and cultured for 48 h at 37°C with 5% CO_2_. Total RNA was extracted from HT‐1080 cells using Trizol reagent after treatment with DMSO, NSC305787, NSC668394, or Erastin for the indicated durations (*n* = 3). Subsequently, sequencing was performed on the Illumina HiSeq X Ten platform (Tsingke, Beijing, China) with paired‐end 150 bp reads. Quality control analysis was performed using FASTQC (v0.11.3). Reads were aligned to the human genome (GRCh38.p2) using STAR (v2.4.2a). Differentially expressed genes with a fold change > 1.5 and a *p*‐value < 0.05, were analyzed using DESeq2 (v1.18.1) in R (v3.4.4). The RNA sequencing results were available at the Gene Expression Omnibus (accession number: SUB14700769 and SUB14675389).

### 4.15 Quantitative RT‐PCR (qPCR)

Total RNA was extracted using Trizol reagent (Thermo, #15596018). RNA was reverse transcribed into cDNA using a transcriptor first‐strand cDNA synthesis kit (Yeasen, #11141ES60). Quantitative RT‐PCR was performed with specific primers and SybrGreen dye (Yeasen, #11201ES08) on a Bio‐rad CFX Maestro real‐time PCR system. The relative expression levels of the target genes were quantified using the 2^−ΔΔCT^ method with GAPDH or Actin as the normalization control. Each qPCR experiment was conducted with three independent biological replicates.

### 4.16 Assessment of Free Radical Scavenging Activity Using the ABTS Assay

The free radical scavenging ability of vitamin C, Fer‐1, NSC305787, and NSC668394 was measured at the indicated concentration using the ABTS free radical scavenging ability detection kit (Solarbio, #BC4775), following the manufacturer's protocol. Each ABTS experiment was conducted with three independent biological replicates.

### 4.17 Extracellular Heme Consumption

Extracellular heme concentrations were measured using a commercial Heme Assay Kit (FineTest, #FN240715) according to the manufacturer's instructions. Culture supernatants were collected from cells treated with DMSO, NSC305787, or NSC668394. Each treatment group comprised nine independent samples.

### 4.18 Mice Organotypic Entorhinal‐Hippocampal Slices (OEHSC) Preparation

Using surgical scissors, decapitate the P3‐P6 neonatal mice and place the heads into a dish (Animal research ethics approval number: SIAT‐IACUC‐20210909‐NS‐NTPZX‐ZXZ‐A1231‐08). Spray the heads with ethanol and transfer them to a laminar flow hood. Using forceps and scissors, remove the skin of mouse head. With a new pair of scissors, cut along the midline of the skull and remove the skull. Transfer the mouse brain to a dish containing pre‐cooled preparation medium (MEM Alpha basic (1x), Gibco) using forceps. Under a dissecting microscope, carefully remove the cerebellum using fine forceps. Separate the left and right hemispheres, remove the midbrain, and trim away excess tissue, leaving the hippocampus‐entorhinal cortex. Transfer this section to a new dish with pre‐cooled preparation medium (MEM, 1% Glutamax, 1% 100x streptomycin‐penicillin, 1% 1 m Tris‐HCl pH 7.4, 1% 1 m HEPES pH 7.3, medium adjusted to pH 7.2–7.4 with sterile 1 m NaOH). Using a spatula, transfer the isolated hippocampus‐entorhinal cortex to a vibratome (McIlwain or Leica VT1200 S) for sectioning. Cut 50 µm thick slices. After sectioning, transfer the slices to a new dish with pre‐cooled preparation medium using a spatula, and carefully separate each slice under a dissecting microscope. Rinse a Millicell insert (Millipore, #PICM03050) with MEM medium and aspirate the medium. Transfer the slices to the Millicell insert, and place the insert into a 6‐well plate containing 1 mL of complete medium (48% MEM, 24% HS, 24% BME, 1% Glutamax, sterile glucose to 1 g/L, 1% 1 m Tris‐HCl pH 7.4, 1% 1 m HEPES pH 7.3 to 10 mm, medium adjusted to pH 7.2–7.4 with sterile 1 m NaOH). Incubate at 37°C. Replace the complete medium after 24 h, and subsequently every other day. After 7–8 days of culture, the slices were ready for OGD/R treatments.

### 4.19 OGD/R Treatment of OEHSC

Prior to OGD/R treatment, pre‐treat the brain slices with 5 µm NSC305787 for 3 h. Transfer all groups designated for OGD/R treatment to a new 6‐well plate containing ice‐cold, serum‐free medium just before OGD/R treatments. Place the plate in a hypoxic chamber, treat with a gas mixture of 95% N_2_ and 5% CO_2_ for 15 min. Simultaneously, place the control group in a laminar flow hood for 15 min. Afterward, incubate all slices in a cell culture incubator (37°C; 5% CO_2_) for 45 min to complete the OGD/R treatment. Replace the medium with complete medium containing 5 µm NSC305787 or 5 µm Fer‐1 (no pre‐treatment), incubate in the cell culture incubator for subsequent experiments. Each treatment group consisted of 8 brain slices derived from four neonatal mice.

### 4.20 Immunofluorescence Staining of Mice Brain Slices

Following treatment under the indicated conditions and durations, 3–15 mouse brain slices were washed three times with PBS and subsequently fixed with 4% paraformaldehyde at 37°C for 15 min. Wash three times with PBS on a shaker for 10 min each. Incubate the slices at room temperature for 1 h with a permeabilization and blocking buffer (5% goat serum + 0.5% Triton X‐100 in PBS), then transfer to a 24‐well plate. Dilute NRF2 or HMOX1 primary antibodies in 5% goat serum + 0.1% Triton X‐100 in PBS at a 300:1 ratio, add 500 µL of this solution to each well, and incubate on a shaker at 4°C for 48 h. Wash with PBS three times on a shaker for 10 min each. Dilute the secondary antibody (Invitrogen, #A11034) at a 1:1000 ratio in 5% goat serum + 0.1% Triton X‐100 in PBS, add 1 µg/mL Hoechst 33342 for nuclei staining, and incubate on a shaker at room temperature in dark for 3 h. Discard the solution, wash three times with PBS for 10 min each, and proceed with confocal imaging.

### 4.21 Cisplatin‐Induced Acute Kidney Injuries in Mice

C57BL/6 mice (6–7 weeks old) were randomly assigned to control (*n* = 6), cisplatin (15 mg/kg, *n* = 6), cisplatin + Fer‐1 (5 mg/kg, *n* = 6), and cisplatin + NSC305787 (2.5 mg/kg, *n* = 6) groups (Animal research ethics approval number: PJ[DW‐2024003‐01]). Cisplatin was dissolved in saline and administered at 3 mg/mL. Pretreatment with NSC305787 was done once at 1 h prior to cisplatin administration. The saline group was injected with the same solvent and saline as the experimental groups. At 0 h, all groups except the saline group were injected with the same concentration of cisplatin. The mice were fed normally and weighed every 24 h and the experiment ended 72 h after cisplatin injection. At the end of the experiment, the mice were anesthetized, and blood samples were collected for biochemical analysis by using sterile techniques by gently pressing on the eyeball to promote blood flow. The mice were then placed on their backs on the operating table. The skin and muscle near the kidneys were carefully incised using a scalpel to expose the kidneys. The kidneys were gently removed using microsurgical instruments, avoiding any damage to the tissue. The left kidney was placed in a light‐protected container with combined fixative (Seville, #G1101) for subsequent pathological examination. The right kidney was placed in fresh saline.

### 4.22 Measurement of Urea Nitrogen and Creatinine

Harvested blood samples were put in room temperature for 1 h. Serum was isolated by centrifugation at 3000 rpm for 15 min at 4°C. Urea nitrogen (Nanjing Jiancheng Bioengineering Institute, #C013‐1‐1) and creatinine (Rayto, #S03079) levels were measured according to the manufacturer's protocols. Each treatment group comprised blood samples obtained from six mice.

### 4.23 Paraffin Embedding and Sectioning of Kidneys

Fix the kidneys in paraformaldehyde (Servicebio, #G1101) solution for at least 24 h. Remove the kidneys from the fixative and place them in a dehydration cassette. Place the cassette in a tissue processor (Leica Histocore PEARL) and dehydrate the tissue through a series of graded alcohols (75% ethanol for 1 h, 85% ethanol for 2 h, 90% ethanol for 1.5 h, 95% ethanol for 1.5 h, and 100% ethanol for 1 h). Next, replace the ethanol with a mixture of half ethanol and half dimethylbenzene for 1 h, followed by dimethylbenzene for 40 min, and then fresh dimethylbenzene for another 40 min. Pour molten paraffin at 65°C into embedding molds and place them in the embedding machine (Leica Histocore Arcadia H), allowing the tissue to infiltrate paraffin in molds I, II, and III sequentially for 1.5, 1.5, and 2 h, respectively. Once the paraffin solidifies, remove the paraffin blocks from the molds and trim them. Section the trimmed paraffin blocks at a thickness of 4 µm using a microtome (Leica HistoCore BIOCUT). Float the sections on 40°C water in a water bath (Leica HI1210), then transfer them to a 60°C oven. After drying, store the sections at room temperature for further use.

### 4.24 Hematoxylin and Eosin (H&E) Staining

Immerse the tissue sections sequentially in dimethylbenzene for 8 min, replace with fresh dimethylbenzene, and repeat twice. Then, place the sections in absolute ethanol for 5 min, replace with fresh absolute ethanol for another 5 min, followed by 85% ethanol for 5 min and 75% ethanol for 5 min. Rinse the sections with distilled water for 2 min. Stain the sections with hematoxylin (Hangzhou Haoke Biotechnology, #HK2053) for 5 min, then differentiate in hydrochloric acid solution (Sinopharm Chemical Reagent, #10011018) for 2 s, and blue in ammonia water solution (Macklin, A801005) for 15–30 s, followed by a water rinse. Dehydrate the sections in 95% ethanol, then stain with eosin for 5–8 s. Sequentially immerse the sections in absolute ethanol solution I for 30 s, absolute ethanol solution II for 2.5 min, absolute ethanol solution III for 2.5 min, dimethylbenzene solution I for 2.5 min, and dimethylbenzene solution II for 2.5 min for clearing. Mount the sections with neutral resin. Examine the slides under a microscope (Motic, #Easyscan), and capture and analyze the images.

### 4.25 Immunohistochemistry

Immerse the tissue sections in dimethylbenzene for 12 min, replace with fresh dimethylbenzene for another 12 min, then sequentially in absolute ethanol for 6 min, 95% ethanol for 6 min, and 85% ethanol for 6 min. Rinse with distilled water. Place the sections in a box filled with EDTA repair solution (pH 8.0, Hangzhou Haoke Biotech, #HKI0003) and perform antigen retrieval in a microwave at medium power for 8 min, pause for 8 min, and then at medium‐low power for 7 min. Allow the slides to cool naturally, then wash them in PBS (pH 7.4) on a shaker three times for 5 min each. Incubate the sections with 3% hydrogen peroxide at room temperature for 25 min to block endogenous peroxidase activity. Wash the slides again in PBS on a shaker three times for 5 min each. Apply 3% BSA to the sections to evenly cover the tissue and block at room temperature for 30 min. After drying the slides, cover the tissue with a diluted primary antibody (NRF2, HMOX1, or 4‐HNE) and incubate in a humid box at 4°C overnight. Wash the slides in PBS on a shaker three times for 5 min each. After slightly drying the sections, apply the appropriate secondary antibody and incubate in dark at room temperature for 50 min. Wash the slides in PBS on a shaker three times for 5 min each. After slightly drying the sections, apply freshly prepared DAB substrate (diluted 20:1 with DAB concentrate) to the tissue, monitoring under a microscope to control the development time until brown‐yellow staining appears, then rinse with distilled water to stop the reaction. Counterstain with hematoxylin for 2–3 min, rinse with distilled water, differentiate in differentiation solution for 2 s, rinse with distilled water, blue in blueing solution for 15–30 s, and rinse with distilled water. Dehydrate the sections sequentially in 75% ethanol for 5 min, 85% ethanol for 5 min, 95% ethanol for 5 min, absolute ethanol for 5 min, dimethylbenzene solution I for 5 min, and dimethylbenzene solution II for 5 min. After removing the sections from dimethylbenzene, air‐dry them slightly and mount with neutral resin.

### 4.26 Reactive Oxygen Species Assay

Intracellular reactive oxygen species (ROS) were detected using the fluorescent probe 2',7'‐dichlorodihydrofluorescein diacetate (DCFH‐DA, Nanjing Jiancheng, #E004‐1‐1). After drug treatment, cells were washed with DMEM and incubated with 10 µm DCFH‐DA in DMEM (Serum‐free) for 25 min at 37°C in the dark. Unincorporated probe was removed by three washes with DMEM (Serum‐free). Cells were then trypsinized and resuspended in DMEM (Serum‐free). Fluorescence intensity of the oxidized product 2',7'‐dichlorofluorescein (DCFH‐DA) was quantified by flow cytometry (excitation/emission: 488 nm/525 nm) to determine ROS levels. Each ROS experiment was conducted with three independent biological replicates.

### 4.27 DHE‐Flow Cytometric Quantification of Superoxide Anion

Superoxide anion levels were assessed with the Beyotime DHE kit (S0063, Beyotime, China). Cells were seeded in 12‐well plates and grown to 70% confluence. After drug treatment, the medium was aspirated and cells were washed twice with ice‐cold PBS. Cells were then loaded with 10 µm DHE prepared in serum‐free medium and incubated at 37°C for 20 min in the dark. After two additional PBS rinses, single‐cell suspensions were immediately analyzed on an Attune NxT Acoustic Focusing Flow Cytometer (Thermo Fisher Scientific); 10 000 events were acquired in the FL2 channel (585 ± 21 nm) and mean fluorescence intensity (MFI) was normalized to the corresponding control. Each DHE experiment was conducted with three independent biological replicates.

### 4.28 Prepare Full Medium or Cystine Deprivation Medium

Full medium (DMEM high‐glucose medium without pheno red; compositions referenced from Procell system, PM150210) was prepared according to the standard formulation. For cystine‐deficient medium, cystine was omitted. The components were dissolved in deionized water, brought to the final volume, and sterilized by filtration through a 0.22 µm filter. Both media were supplemented with 10% fetal bovine serum and 1% penicillin‐streptomycin before use.

### 4.29 Confocal Microscopy Imaging

Microscopy imaging was performed using Olympus FV3000 or SpinSR equipped with a definite focus system, using either a 60× oil‐immersion lens (NA 1.42) or a 40× oil‐immersion lens (NA 1.30). General acquisition settings include a Z step size of 0.5–2 µm. Live cells were imaged within a heated, humidified incubation chamber with CO_2_ control. Fixed cells and tissues were imaged in room temperature.

### 4.30 Molecular Docking by DiffDock‐L

DiffDock‐L (https://neurosnap.ai/service/DiffDock‐L) is used for molecular docking stimulation between full length Ezrin (PDB Entry: 4RM9) and NSC305787 (PubChem CID: 470998) or NSC668394 (PubChem CID: 381594). Top 9–10 results were shown.

### 4.31 Image Analysis and Processing

Confocal microscopy images were analyzed and processed using Fiji (http://fiji.sc).

### 4.32 Quantification and Statistical Analysis

Data were analyzed using GraphPad Prism 10.3.1 software. Mean, standard error of the mean (SEM), and P‐values were calculated using the Student's unpaired t‐test with Welch's correction, Mann‐Whitney U test, One‐way ANOVA, or Two‐way ANOVA. When ANOVA results were significant, post‐hoc comparisons were performed with Tukey's multiple comparisons test. Significance levels were indicated as *p < 0.05, **p < 0.01, ***p < 0.001, ****p < 0.0001; n.s., not significant. Unless otherwise stated in the figure legends, all experiments were performed independently three times. Schematic illustrations were created with BioRender.com.

## Author Contributions

**Junqi Huang, Ting Gang Chew, Yunmiao Guo, and Lijuan Pang**: conceptualization, funding acquisition, and writing. **Menghao Qiao, Liqun Zhou, Minhua Zhou, Yu Fang, Haiying Mai, Lingbo Cao, Kun Xu, Yuan Sang, Minyi Chen, Jiewei Huang, Peiyi Huang, Zhipeng Yan, Chao Wang, Zhangshuai Dai, Dichun Huang, and Ronghan He**: experiments. **Menghao Qiao, Liqun Zhou, and Junqi Huang**: data analysis.

## Conflicts of Interest

A patent related to this work has been issued (CN117462547B). The authors declare no other competing interests.

## Supporting information




**Supporting File 1**: advs73460‐sup‐0001‐SuppMat.docx.

**Supporting File 2**: advs73460‐sup‐0002‐TableS1.xlsx.

**Supporting File 3**: advs73460‐sup‐0003‐DataFile.ai.

## Data Availability

Requests for further information and resources should be directed to and will be fulfilled by the corresponding authors. Materials generated in this study are available upon request to the corresponding authors without restrictions. The RNA sequencing data have been deposited at the Gene Expression Omnibus as (accession number: SUB14700769 and SUB14675389) and are publicly available as of the date of publication. This paper does not report original code. Any additional information required to reanalyze the data reported in this paper is available from the corresponding authors upon request.
