# Supplementary material for: ERM Inhibition Confers Ferroptosis Resistance through ROS‐Induced NRF2 Signaling
Source: Adv Sci (Weinh). 2026 Jan 27;13(16):e13310. doi: 10.1002/advs.202513310 (PMC13042784; doi:10.1002/advs.202513310)
Supplement: Supplementary file 1 — Supporting File 1: advs73460‐sup‐0001‐SuppMat.docx. [file ADVS-13-e13310-s001.docx]

**Supplementary Figure 1 | Screening of cytoskeleton-targeting compounds identifies small-molecule ferroptosis inhibitors.**

**a** Cell death measurement of HT-1080 cells treated with Erastin (5 μM) in combination with NSC305787 (2 μM), NSC668394 (2 μM), Pfn1-IN-1 (2 μM), NP-G2-004 (2 μM), Y-27632 (2 μM), SMIFH2 (2 μM) or Benproperine phosphate (2 μM) for 18 h. Dead cells were labeled with Propidium iodide.

**b** Cell death measurement of HT-1080 cells treated with increasing concentrations of NSC305787 in combination with Erastin (5 μM) for 25 h. Dead cells were labeled with Propidium iodide.

**c** Cell death measurement of HT-1080 cells treated with increasing concentrations of NSC668394 in combination with Erastin (5 μM) for 29.5 h. Dead cells were labeled with Propidium iodide.

**d** Cell death measurement of HT-1080 cells treated with increasing concentrations of Pfn1-IN-1 in combination with Erastin (5 μM) for 16 h. Dead cells were labeled with Propidium iodide.

**e** Cell death measurement of HT-1080 cells treated with increasing concentrations of NP-G2-044 in combination with Erastin (5 μM) for 20 h. Dead cells were labeled with Propidium iodide.

**f** Cell death measurement of HT-1080 cells treated with increasing concentrations of Y-27632 in combination with Erastin (5 μM) for 16 h. Dead cells were labeled with Propidium iodide.

**g** Cell death measurement of HT-1080 cells treated with increasing concentrations of SMIFH2 in combination with Erastin (5 μM) for 16 h. Dead cells were labeled with Propidium iodide.

**h** Cell death measurement of HT-1080 cells treated with increasing concentrations of Benproperine phosphate in combination with Erastin (5 μM) for 16 h. Dead cells were labeled with Propidium iodide.

Data and error bars are mean ± SEM, *n =* 3 biologically independent experiments in b-h. *p < 0.05; **p < 0.01; ***p < 0.001; ****p < 0.0001; n.s., not significant. All P values were calculated using a one-way analysis of variance (ANOVA).

**Supplementary Figure 2 | ERM inhibitors NSC305787 and NSC668394 affect HT-1080 cell proliferation.**

**a-b** Cell viability assessed by CCK-8 assay in HT-1080 cells treated with DMSO, or NSC305787 (2 μM) at the indicated concentration for 24 h (a) or 48 h (b).

**c-d** Cell viability assessed by CCK-8 assay in HT-1080 cells treated with DMSO, or NSC668394 (5 μM) at the indicated concentration for 24 h (c) or 48 h (d).

**e-g** Cell death measurement of HT-1080 cells treated with increasing concentrations of DMSO or NSC305787 for 24 h (e), 48 h (f), or 72 h (g). Dead cells were labeled with Propidium iodide.

**h-j** Cell death measurement of HT-1080 cells treated with increasing concentrations of DMSO or NSC668394 for 24 h (h), 48 h (i), or 72 h (j). Dead cells were labeled with Propidium iodide.

Data and error bars are mean ± SEM, *n =* 3 biologically independent experiments in a-j. *p < 0.05; **p < 0.01; ***p < 0.001; ****p < 0.0001; n.s., not significant. All P values were calculated using a one-way analysis of variance (ANOVA).

**Supplementary Figure 3 | ERM inhibitors modulate ferroptosis across multiple cell lines.**

**a** Representative Hoechst and PI staining images of HT-1080 cells treated with DMSO, Erastin (5 μM), NSC305787 (2 μM), NSC668394 (5 μM), or their combinations. Images were acquired as SUM projections using a cell imaging multimode fluorescence plate reader with a 4x objective, capturing data through DAPI and Texas Red channels. Each image shows approximately 300 cells.

**b** Cell death measurement of OVCAR-8 cells treated with Erastin (5 μM) in combination with Fer-1 (2 μM), and NSC305787 at the indicated concentration for 24 h. Dead cells were labeled with Propidium iodide.

**c** Cell death measurement of 293T cells treated with Erastin (5 μM) and NSC305787 (0.5 and 1.5 μM, pre-treated 3 h) for 21 h. Dead cells were labeled with Propidium iodide.

**d** Cell death measurement of HeLa cells treated with Erastin (10 μM) and NSC305787 (0.5 and 1.5 μM, pre-treated 3 h) for 25 h. Dead cells were labeled with Propidium iodide.

**e** Cell death measurement of OVCAR-8 cells treated with Erastin (5 μM) in combination with Fer-1 (2 μM), and NSC668394 at the indicated concentration for 24 h. Dead cells were labeled with Propidium iodide.

**f** Cell death measurement of 293T cells treated with Erastin (10 μM) and increasing concentrations across a dose range of NSC668394 (1.25, 2.5, 5, 10 and 20 μM) for 24 h. Dead cells were labeled with Propidium iodide.

**g** Cell death measurement of HeLa cells treated with Erastin (10 μM) and increasing concentrations across a dose range of NSC668394 (1.25, 2.5, 5, 10 and 20 μM) for 24 h. Dead cells were labeled with Propidium iodide.

Data and error bars are mean ± SEM, *n =* 3 biologically independent experiments in b-g. *p < 0.05; **p < 0.01; ***p < 0.001; ****p < 0.0001; n.s., not significant. All P values were calculated using a one-way or two-way analysis of variance (ANOVA).

**Supplementary Figure 4 | ERM inhibitors attenuate cystine deprivation-induced ferroptosis in HT-1080 cells.**

**a** Cell death measurement of HT-1080 cells treated with NSC305787 (2 μM) under full medium or cystine deprivation for 24 h. Dead cells were labeled with Propidium iodide.

**b** Cell death measurement of HT-1080 cells treated with NSC305787 (2 μM, pre-treated 3 h) and RSL3 (0.25 and 0.5 μM) for 4.5 h. Dead cells were labeled with Propidium iodide.

**c** Cell death measurement of HT-1080 cells treated with NSC305787 (2 μM, pre-treated 3 h) and ML210 (0.25 and 0.5 μM) for 4.5 h. Dead cells were labeled with Propidium iodide.

**d** Cell death measurement of HT-1080 cells treated with NSC668394 (5 μM) under full medium or cystine deprivation for 24 h. Dead cells were labeled with Propidium iodide.

**e** Cell death measurement of HT-1080 cells treated with NSC668394 (5 μM) and RSL3 (2 μM) for 12 h. Dead cells were labeled with Propidium iodide.

**f** Cell death measurement of HT-1080 cells treated with NSC668394 (5 μM) and ML210 (5 μM) for 12 h. Dead cells were labeled with Propidium iodide.

Data and error bars are mean ± SEM, *n =* 3 biologically independent experiments in a-f. *p < 0.05; **p < 0.01; ***p < 0.001; ****p < 0.0001; n.s., not significant. All P values were calculated using a one-way or two-way analysis of variance (ANOVA).

**Supplementary Figure 5 | Antioxidant activity of ERM inhibitors and their effects on other forms of programmed cell death.**

**a** Schematic illustration of the ABTS radical scavenging assay.

**b** ABTS radical scavenging activity of Vitamin C (1 mM, positive control), Fer-1 (1 mM), NSC305787 (1 mM), and NSC668394 (1 mM).

**c** Cell death measurement of HT-1080 cells treated with MG-132 (1 μM, apoptosis inducer) in combination with NSC305787 (2 μM) or Z-VAD (40 μM, apoptosis inhibitor) for 22 h. Dead cells were labeled with Propidium iodide.

**d** Cell death measurement of HT-1080 cells treated with MG-132 (1 μM) in combination with increasing concentrations NSC668394 (2.5, 5 and 10 μM) or Z-VAD (40 μM) for 24 h. Dead cells were labeled with Propidium iodide.

**e** Cell death measurement of HT-1080 cells treated with CuCl_2_ (1.5 μM) and Elesclomol (1.5 μM, a cuproptosis inducer combination) in combination with NSC305787 (2 μM, pre-treated 2.5 h) or TTM (20 μM, cuproptosis inhibitor) for 13 h. Dead cells were labeled with Propidium iodide.

**f** Cell death measurement of HT-1080 cells treated with CuCl_2_ (1.5 μM) and Elesclomol (1.5 μM) in combination with NSC668394 (10 μM) or TTM (20 μM) for 13 h. Dead cells were labeled with Propidium iodide.

Data and error bars are mean ± SEM, *n =* 3 biologically independent experiments in b-f. *p < 0.05; **p < 0.01; ***p < 0.001; ****p < 0.0001; n.s., not significant. All P values were calculated using a one-way or two-way analysis of variance (ANOVA).

**Supplementary Figure 6 | Predicted ERM protein structures and ERM mRNA and protein expression in HT-1080 Cells.**

**a** AlphaFold3-predicted 3D structures of Ezrin, Radixin, and Moesin. Purple highlights indicate the antigen sequence (GRDKYKpTLRQIR) used to generate the pERM antibody. Pairwise structural similarity scores (TM-score): Ezrin vs Radixin = 0.82, Ezrin vs Moesin = 0.75, Radixin vs Moesin = 0.85.

**b-c** mRNA (b) and protein (c) expression levels of Ezrin, Radixin, and Moesin in HT-1080 cells obtained from The Human Protein Atlas (https://www.proteinatlas.org/).

**d-f** Protein expression levels of Ezrin (d), Radixin (e), and Moesin (f) in HT-1080 cells.

**g** Quantification of ERM protein levels from d-f.

Data and error bars are mean ± SEM, *n =* 3 biologically independent experiments in g. *p < 0.05; **p < 0.01; ***p < 0.001; ****p < 0.0001; n.s., not significant. All P values were calculated using a one-way or two-way analysis of variance (ANOVA).

**Supplementary Figure 7 | Genetic depletion of ERM proteins in HT-1080 cells and their effect on pERM level.**

**a-c** Knockdown efficiency of Ezrin (a), Radixin (b), and Moesin (c) using two independent shRNAs, validated by qPCR.

**d** Cell death measurement of shNC and shEzrin cells under full medium or cystine deprivation for 36 h. Dead cells were labeled with Propidium iodide.

**e** Cell death measurement of shNC and shEzrin cells treated with RSL3 (0.5 and 1 μM) for 6 h. Dead cells were labeled with Propidium iodide.

**f** Cell death measurement of shNC and shEzrin cells treated with ML210 (0.5 and 1 μM) for 6.5 h. Dead cells were labeled with Propidium iodide.

**g** Cell death measurement of shNC and shRadixin cells treated with RSL3 (0.25, 0.5 and 1 μM) for 7 h. Dead cells were labeled with Propidium iodide.

**h** Cell death measurement of shNC and shMoesin cells treated with RSL3 (0.25, 0.5 and 1 μM) for 7 h. Dead cells were labeled with Propidium iodide.

**i-j** Western blot analysis and quantification of Ezrin protein levels in shNC and ERM-knockdown cells.

**k-l** Western blot analysis and quantification of Radixin protein levels in shNC and ERM-knockdown cells.

**m-n** Western blot analysis and quantification of Moesin protein levels in shNC and ERM-knockdown cells.

Data and error bars are mean ± SEM, *n =* 3 biologically independent experiments in a-h, j, l, and n. *p < 0.05; **p < 0.01; ***p < 0.001; ****p < 0.0001; n.s., not significant. All P values were calculated using a one-way or two-way analysis of variance (ANOVA).

**Supplementary Figure 8 | Overexpression of ERM proteins modulates ferroptosis sensitivity.**

**a** Overexpression of Ezrin wild-type (WT)-mCherry in HT-1080 cells validated by qPCR.

**b** Western blot analysis using an anti-Ezrin antibody in HT-1080 cells overexpressing vector control (backbone plasmid), Ezrin WT, Radixin WT, or Moesin WT. All constructs are mCherry-tagged.

**c-d** Quantification of endogenous (c) and total Ezrin (d) protein levels from b.

**e** Overexpression of Radixin WT-mCherry in HT-1080 cells validated by qPCR.

**f** Western blot analysis using an anti-Radixin antibody in HT-1080 cells overexpressing vector control, Ezrin WT, Radixin WT, or Moesin WT. All constructs are mCherry-tagged.

**g-h** Quantification of endogenous (g) and total Radixin (h) protein levels from f.

**i** Overexpression of Moesin WT-mCherry in HT-1080 cells validated by qPCR.

**j** Western blot analysis using an anti-Moesin antibody in HT-1080 cells overexpressing vector control, Ezrin WT, Radixin WT, or Moesin WT. All constructs are mCherry-tagged.

**k-l** Quantification of endogenous (k) and total Moesin (l) protein levels from j.

**m-o** Cell death measurement of HT-1080 cells overexpressing vector control, Ezrin WT (m), Radixin WT (n), or Moesin WT (o) following treatment with Erastin (5 μM) for the indicated durations. Dead cells were labeled with SYTOX-Green.

**p** Cell death measurement of Hep G2 cells overexpressing vector control or Ezrin WT under full medium or cystine deprivation for 105 h. Dead cells were labeled with SYTOX-Green.

**q** Cell death measurement of Hep G2 cells overexpressing vector control or Ezrin WT treated with RSL3 (10 and 15 μM) for 5 h. Dead cells were labeled with SYTOX-Green.

**r** Cell death measurement of Hep G2 cells overexpressing vector control or Ezrin WT treated with ML210 (10 and 15 μM) for 5 h. Dead cells were labeled with SYTOX-Green.

Data and error bars are mean ± SEM, *n =* 3 biologically independent experiments in a, c-e, g-i and k-r. *p < 0.05; **p < 0.01; ***p < 0.001; ****p < 0.0001; n.s., not significant. All P values were calculated using a one-way or two-way analysis of variance (ANOVA).

**Supplementary Figure 9** **| Binding prediction, ERM protein expression, and functional validation of ERM phosphorylation.**

**a** Molecular docking of NSC305787 or NSC668394 with Ezrin protein (PDB Entry: 4RM9) by DiffDock-L. Left image: top 9-10 best predictions are shown simultaneously; Middle image: enlarged image showing the distance of the closest prediction between NSC305787 or NSC668394 with Thr567 of Ezrin; Right table: metrics and scores of the predictions ordered from best to worst, with red color showing the prediction data of the enlarged image.

**b-c** Western blot analysis and quantification of Ezrin protein levels in HT-1080 cells treated with DMSO, NSC305787 (2 μM) or NSC668394 (5 μM) for 10 h.

**d-e** Western blot analysis and quantification of Radixin protein levels in HT-1080 cells treated with DMSO, NSC305787 (2 μM) or NSC668394 (5 μM) for 10 h.

**f-g** Western blot analysis and quantification of Moesin protein levels in HT-1080 cells treated with DMSO, NSC305787 (2 μM) or NSC668394 (5 μM) for 10 h.

**h-j** Western blot analysis and quantification of endogenous pERM (i) and total pERM (endogenous pERM and phosphorylated Radixin-mCherry) (j) protein levels in HT-1080 cells overexpressing vector control or Radixin WT. All constructs are mCherry-tagged.

**k-m** Western blot analysis and quantification of endogenous pERM (l) and total pERM (endogenous pERM and phosphorylated Moesin-mCherry) (m) protein levels in HT-1080 cells overexpressing vector control or Moesin WT. All constructs are mCherry-tagged.

**n** Sequencing results confirming the T567A and T567D Ezrin point mutations in overexpression plasmids.

**o** Cell death measurement of HT-1080 cells treated with SB-633825 (2, 5 and 10 μM, pre-treated 14 h) under full medium or cystine deprivation for 24 h. Dead cells were labeled with Propidium iodide.

**p** Cell death measurement of HT-1080 cells treated with SLK/STK10-IN-1 (20, 50 and 100 μM, pre-treated 14 h) under full medium or cystine deprivation for 24 h. Dead cells were labeled with Propidium iodide.

Data and error bars are mean ± SEM, *n =* 3 biologically independent experiments in c, e, g, i-j, l-m and o-p. *p < 0.05; **p < 0.01; ***p < 0.001; ****p < 0.0001; n.s., not significant. All P values were calculated using a one-way or two-way analysis of variance (ANOVA).

**Supplementary Figure 10 | RNA sequencing analysis of HT-1080 cells treated with ERM inhibitors or erastin.**

**a** Graph showing number of differentially expressed genes from RNA sequencing with the indicated treatments.

**b** KEGG annotation analysis of differentially expressed genes in HT-1080 cells treated with Erastin for 8 or 13 h, showing enrichment in the regulation of actin cytoskeleton (*n* = 3).

**c** Gene Set Enrichment Analysis (GSEA) plots in HT-1080 cells treated with NSC305787, showing enrichment of gene sets related to: response to reactive oxygen species, ferroptosis, cortical actin cytoskeleton, and cytoskeleton protein binding (*n* = 3).

**d** KEGG analysis indicated that the differentially up expressed genes in HT-1080 cells treated with NSC668394 (5 μM) were enriched in ferroptosis. The number of genes represented in each pathway was indicated by point size, and the *p*-value was shown by point color (*n* = 3).

**e** GO analysis (Cellular Component) indicated that the differentially expressed genes in HT-1080 cells treated with NSC668394 (5 μM) were enriched in actin cytoskeleton and actin-based cell projection. The number of genes represented in each pathway was indicated by point size, and the *p*-value was shown by point color (*n* = 3).

**f** GO analysis (Cellular Component) indicated that the differentially down expressed genes in HT-1080 cells treated with NSC668394 (5 μM) were enriched in cortical actin cytoskeleton. The number of genes represented in each pathway was indicated by point size, and the *p*-value was shown by point color (*n* = 3).

**g** GO analysis (Biological Process) indicated that the differentially up expressed genes in HT-1080 cells treated with NSC668394 (5 μM) were enriched in positive regulation of actin filament bundle assembly. The number of genes represented in each pathway was indicated by point size, and the *p*-value was shown by point color (*n* = 3).

**Supplementary Figure 11 | RNA sequencing analysis of HT-1080 cells treated with NSC305787 and the effects of ERM inhibition and LatA on the actin cytoskeleton.**

**a** KEGG annotation analysis indicated that the differentially expressed genes in HT-1080 cells treated with NSC305787 (2 μM) were enriched in the regulation of actin cytoskeleton (*n* = 3).

**b** KEGG annotation analysis indicated that the differentially up expressed genes in HT-1080 cells treated with NSC305787 (2 μM) were enriched in the regulation of actin cytoskeleton (*n* = 3).

**c** KEGG annotation analysis indicated that the differentially down expressed genes in HT-1080 cells treated with NSC305787 (2 μM) were enriched in the regulation of actin cytoskeleton (*n* = 3).

**d** GO analysis (Cellular Component) indicated that the differentially expressed genes in HT-1080 cells treated with NSC305787 (2 μM) were enriched in actin cytoskeleton. The number of genes represented in each pathway was indicated by point size, and the *p*-value was shown by point color (*n* = 3).

**e** KEGG pathway analysis of differentially expressed genes in HT-1080 cells treated with NSC305787 (2 μM), showing enrichment in ferroptosis and regulation of actin cytoskeleton. The number of genes represented in each pathway was indicated by point size, and the *p*-value was shown by point color (*n* = 3).

**f** Gene Ontology (GO) analysis (Biological Process, GO_BP) of the dataset generated from HT-1080 cells treated with NSC305787 (2 μM), showing enrichment in response to reactive oxygen species and actin nucleation (*n* = 3).

**g** Representative confocal microscopy images of phalloidin-stained HT-1080 cells treated with DMSO, NSC305787 (2 μM), or NSC668394 (5 μM) for 10 h. Cells treated with NSC305787 or NSC668394 exhibit reduced lamellipodia and membrane ruffles. Images were acquired as SUM projections using a confocal microscopy with a 60x objective, capturing data through FITC channels. The image above shows an enlarged view of the area within the yellow box. Confocal imaging was repeated twice, with each including 16-21 cells.

**h** Time-lapse imaging of LifeAct-mScarletI-expressed HT-1080 cells following LatA treatment. Confocal imaging was repeated twice (*n* > 30). The time point marked as 0 min represents the start of the movie, with a ~15-30 min gap between chemical treatment and the beginning of the imaging.

**Supplementary Figure 12** **| F-actin disassembly attenuates erastin-induced ferroptosis sensitivity across multiple cell lines.**

**a** Cell death measurement of HT-1080 cells treated with LatA at the indicated concentration under full medium or cystine deprivation for 34 h. Dead cells were labeled with Propidium iodide.

**b** Cell death measurement of HT-1080 cells treated with LatA (0.05, 0.1 and 0.2 μg/mL) and RSL3 (0.5 μM) for 5.5 h. Dead cells were labeled with Propidium iodide.

**c** Cell measurement of HeLa cells treated with LatA (0.15 and 0.2 μg/mL) and Erastin (10 μM) for 24 h. Dead cells were labeled with Propidium iodide.

**d** Cell death measurement of 293T cells treated with LatA (0.15 μg/mL) and Erastin (5 μM) for 17 h. Dead cells were labeled with Propidium iodide.

**e** Cell death measurement of 4T1 cells treated with LatA (0.05 μg/mL) and Erastin (5 μM) for 18 h. Dead cells were labeled with Propidium iodide.

**f** Cell death measurement of 3T3 cells treated with LatA (0.025, 0.05 and 0.2 μg/mL) and Erastin (5 μM) for 12 h. Dead cells were labeled with Propidium iodide.

**g** Cell death measurement of HT-1080 cells treated with CK-666 (50, 100 and 200 μM) and Erastin (5 μM) for 19 h. Dead cells were labeled with Propidium iodide.

**h** Cell death measurement of HT-1080 cells with CK-666 (100, 150 and 200 μM) under full medium or cystine deprivation for 25 h. Dead cells were labeled with Propidium iodide.

**i** Cell death in HT-1080 cells with CK-666 (100, 150 and 200 μM) and RSL3 (2 μM) for 12 h. Dead cells were labeled with Propidium iodide.

Data and error bars are mean ± SEM, *n =* 3 biologically independent experiments in a-i. *p < 0.05; **p < 0.01; ***p < 0.001; ****p < 0.0001; n.s., not significant. All P values were calculated using a one-way or two-way analysis of variance (ANOVA).

**Supplementary Figure 13 | Knockdown of INF2 attenuates erastin-induced ferroptosis in HT-1080 cells.**

**a** Relative INF2 mRNA levels in cells transfected with control siRNA (siNC) or INF2 siRNA (siINF2) for 48 h, measured by qPCR.

**b** Western blot analysis of INF2 protein levels after 48 h transfection with the indicated siNC and siINF2 cells.

**c** Quantification of western blot from b.

**d** Cell death measurement of siINF2-transfected cells treated with Erastin (10 μM) for 22 h. Dead cells were labeled with Propidium iodide.

**e-f** Western blot analysis and quantification of INF2 protein levels in cells treated with DMSO, NSC305787 (2 μM) or NSC668394 (5 μM) for 10 h.

**g-h** Western blot analysis and quantification of INF2 protein levels in HT-1080 cells treated with Erastin (5 μM) alone, or in combination with NSC305787 (2 μM), or NSC668394 (5 μM) for 10 h.

**i** INF2 knockdown efficiency confirmed by qPCR using two independent shRNAs.

**j-k** Western blot analysis and quantification of INF2 protein levels in cells transduced with control (shNC) or INF2-targeting shRNAs (shINF2).

**l** Cell death measurement of shNC and shINF2 cells treated with Erastin (2.5 and 5 μM) for 17.5 h. Dead cells were labeled with Propidium iodide.

Data and error bars are mean ± SEM, *n =* 3 biologically independent experiments in a, c-d, f, h-i and k-l. *p < 0.05; **p < 0.01; ***p < 0.001; ****p < 0.0001; n.s., not significant. All P values were calculated using a one-way or two-way analysis of variance (ANOVA).

**Supplementary Figure 14** **| NRF2 inhibition compromises ferroptosis protection by ERM inhibition and actin perturbation.**

**a** Cell death measurement of HT-1080 cells treated with the indicated combination of Brusatol (1 μM, pre-treated 13.5 h), Erastin (5 μM), and Fer-1 (2 and 5 μM) for 46 h. Dead cells were labeled with Propidium iodide.

**b** Cell death measurement of HT-1080 cells treated with the indicated combination of Brusatol (1 μM, pre-treated 13.5 h), Erastin (5 μM), and Lip-1 (2 and 5 μM) for 46 h. Dead cells were labeled with Propidium iodide.

**c** Cell death measurement of HT-1080 cells treated with the indicated combination of Brusatol (1 μM, pre-treated 13.5 h), Erastin (5 μM), and (100 and 200 μM) for 46 h. Dead cells were labeled with Propidium iodide.

**d** Cell death measurement of HT-1080 cells treated with the indicated combination of Brusatol (1 μM, pre-treated 11 h), Erastin (5 μM), and Fer-1 (2 and 5 μM) for 36 h. Dead cells were labeled with Propidium iodide.

**e** Cell death measurement of HT-1080 cells treated with the indicated combination of Brusatol (1 μM, pre-treated 11 h), Erastin (5 μM), and Lip-1 (2 and 5 μM) for 36 h. Dead cells were labeled with Propidium iodide.

**f** Cell death measurement of HT-1080 cells treated with the indicated combination of Brusatol (1 μM, pre-treated 11 h), Erastin (5 μM), and DFO (100 and 200 μM) for 36 h. Dead cells were labeled with Propidium iodide.

**g** Cell death measurement of HT-1080 cells treated with Erastin (5 μM) and increasing concentrations Brusatol (0.125, 0.25, 0.5, 1 and 2 μM, pre-treated 12 h) or Fer-1 (2 μM) for 24 h. Dead cells were labeled with Propidium iodide.

**h** Cell death measurement of HT-1080 cells treated with Erastin (5 μM) and increasing concentrations ML385 (1, 2, 4, 6, 8 and 10 μM, pre-treated 4 h) or Fer-1 (2 μM) for 11.5 h. Dead cells were labeled with Propidium iodide.

**i** Cell death measurement of HT-1080 cells treated with the indicated combination of ML385 (pre-treated 3 h), Erastin (5 μM), and NSC305787 (2 μM) for 24 h. Dead cells were labeled with Propidium iodide.

**j** Cell death measurement of HT-1080 cells treated with the indicated combination of ML385 (pre-treated 3 h), Erastin (5 μM), and NSC668394 (5 μM) for 72 h. Dead cells were labeled with Propidium iodide.

**k-l** Western blot analysis and quantification of total NRF2 protein levels in HT-1080 cells treated with Erastin (5 μM) alone, or in combination with LatA (0.05 μg/mL) for 8 h.

**m** Cell death measurement of HT-1080 cells treated with the indicated combination of Brusatol (1 μM, pre-treated 11 h), Erastin (5 μM), and LatA (0.05 and 0.1 μg/mL) for 30 h. Dead cells were labeled with Propidium iodide.

**n** Cell death measurement of HT-1080 cells treated with the indicated combination of Brusatol (1 μM, pre-treated 11 h), Erastin (5 μM), and CK-666 (100, 150 and 200 μM) for 61.5 h. Dead cells were labeled with Propidium iodide.

**o** Cell death measurement of shNC or shINF2 cells treated with the indicated combination of Brusatol (1 μM, pre-treated 10.5 h) and Erastin (5 μM) for 36 h. Dead cells were labeled with Propidium iodide.

Data and error bars are mean ± SEM, *n =* 3 biologically independent experiments in a-j and l-o. *p < 0.05; **p < 0.01; ***p < 0.001; ****p < 0.0001; n.s., not significant. All P values were calculated using a one-way or two-way analysis of variance (ANOVA).

**Supplementary Figure 15 | The actin cytoskeleton regulates NRF2 nuclear translocation.**

**a** Immunofluorescence staining of NRF2 and Hoechst in HT-1080 cells treated with DMSO or LatA (0.05 μg/mL) for 10 h. Images were acquired as SUM projections using a confocal microscopy with a 60x objective, capturing data through DAPI and mCherry channels. Confocal imaging was repeated twice, with each including 20-25 cells.

**b** Quantification of nuclear NRF2 mean intensity from a (*n* = 15).

**c** Immunofluorescence staining of NRF2, phalloidin, and Hoechst in HT-1080 cells treated with jasplakinolide (40 nM) for 10 h. Images were acquired as SUM projections using a confocal microscopy with a 60x objective, capturing data through DAPI and mCherry channels. Confocal imaging was repeated twice, with each including 26-30 cells.

**d** Quantification of nuclear NRF2 mean intensity from e (*n* = 15).

**e-f** Western blot analysis and quantification of NRF2 protein levels in the cytoplasm and nucleus of HT-1080 cells treated with DMSO or LatA (0.05 μg/mL) for 10 h.

**g-h** Western blot analysis and quantification of NRF2 protein levels in the cytoplasm and nucleus of HT-1080 cells treated with DMSO or jasplakinolide (40 nM) for 10 h.

Data and error bars are mean ± SEM, *n =* 3 biologically independent experiments in j and l. *p < 0.05; **p < 0.01; ***p < 0.001; ****p < 0.0001; n.s., not significant. All P values were calculated using a one-way or two-way analysis of variance (ANOVA).

**Supplementary Figure 16** **| HMOX1 inhibition compromises ferroptosis protection by ERM inhibition and actin perturbation.**

**a** qPCR analysis of KEAP1 mRNA levels in HT-1080 cells treated with DMSO, NSC305787 (2 μM) or NSC668394 (5 μM) for 8 h.

**b** qPCR analysis of SLC7A11 mRNA levels in HT-1080 cells treated with DMSO, NSC305787 (2 μM) or NSC668394 (5 μM) for 8 h.

**c** qPCR analysis of HMOX1 mRNA levels in HT-1080 cells treated with DMSO, NSC305787 (2 μM) or NSC668394 (5 μM) for 8 h.

**d** qPCR analysis of GCLM mRNA levels in HT-1080 cells treated with DMSO, NSC305787 (2 μM) or NSC668394 (5 μM) for 8 h.

**e** qPCR analysis of PRDX1 mRNA levels in HT-1080 cells treated with DMSO, NSC305787 (2 μM) or NSC668394 (5 μM) for 8 h.

**f** qPCR analysis of FTH1 mRNA levels in HT-1080 cells treated with DMSO, NSC305787 (2 μM) or NSC668394 (5 μM) for 8 h.

**g** qPCR analysis of GPX4 mRNA levels in HT-1080 cells treated with DMSO, NSC305787 (2 μM) or NSC668394 (5 μM) for 8 h.

**h** ABTS radical scavenging activity of Vitamin C (1 mM, positive control), Fer-1 (1 mM), Brusatol (1 mM), ML385 (1 mM) and ZnPP (1 mM).

**i** Cell death measurement of HT-1080 cells treated with the indicated combination of ZnPP (10 μM, pre-treated 4 h), Erastin (5 μM), and Fer-1 (2 and 5 μM) for 46 h. Dead cells were labeled with Propidium iodide.

**j** Cell death measurement of HT-1080 cells treated with the indicated combination of ZnPP (10 μM, pre-treated 5 h), Erastin (5 μM), and Lip-1 (2 and 5 μM) for 46 h. Dead cells were labeled with Propidium iodide.

**k** Cell death measurement of HT-1080 cells treated with the indicated combination of ZnPP (10 μM, pre-treated 4 h), Erastin (5 μM), and DFO (100 and 200 μM) for 46 h. Dead cells were labeled with Propidium iodide.

**l** Cell death measurement of HT-1080 cells treated with the indicated combination of ZnPP (10 μM, pre-treated 5 h), Erastin (5 μM), and LatA (0.05 and 0.1 μg/mL) for 36 h. Dead cells were labeled with Propidium iodide.

**m** Cell death measurement of HT-1080 cells treated with the indicated combination of ZnPP (10 μM, pre-treated 6.5 h), Erastin (5 μM), and CK-666 (150 and 200 μM) for 42 h. Dead cells were labeled with Propidium iodide.

**n** Cell death measurement of shNC or shINF2 cells treated with the indicated combination of ZnPP (10 μM, pre-treated 5.5 h) and Erastin (5 μM) for 41 h. Dead cells were labeled with Propidium iodide.

Data and error bars are mean ± SEM, *n =* 3 biologically independent experiments in a-n. *p < 0.05; **p < 0.01; ***p < 0.001; ****p < 0.0001; n.s., not significant. All P values were calculated using a one-way or two-way analysis of variance (ANOVA).

**Supplementary Figure 17 | Transient ERM inhibition induces sustained cell protection against erastin-induced ferroptosis.**

**a** qPCR analysis of KEAP1 mRNA levels in HT-1080 cells treated with DMSO or NSC305787 (2 μM) for 8 h, followed by removal of NSC305787 and incubation for an additional 20 h.

**b** qPCR analysis of NRF2 mRNA levels in HT-1080 cells treated with DMSO or NSC305787 (2 μM) for 8 h, followed by removal of NSC305787 and incubation for an additional 20 h.

**c** qPCR analysis of HMOX1 mRNA levels in HT-1080 cells treated with DMSO or NSC305787 (2 μM) for 8 h, followed by removal of NSC305787 and incubation for an additional 20 h.

**d** qPCR analysis of SLC7A11 mRNA levels in HT-1080 cells treated with DMSO or NSC305787 (2 μM) for 8 h, followed by removal of NSC305787 and incubation for an additional 20 h.

**e** qPCR analysis of KEAP1 mRNA levels in HT-1080 cells treated with DMSO or NSC668394 (5 μM) for 8 h, followed by removal of NSC305787 and incubation for an additional 20 h.

**f** qPCR analysis of NRF2 mRNA levels in HT-1080 cells treated with DMSO or NSC668394 (5 μM) for 8 h, followed by removal of NSC305787 and incubation for an additional 20 h.

**g** qPCR analysis of HMOX1 mRNA levels in HT-1080 cells treated with DMSO or NSC668394 (5 μM) for 8 h, followed by removal of NSC305787 and incubation for an additional 20 h.

**h** qPCR analysis of SLC7A11 mRNA levels in HT-1080 cells treated with DMSO or NSC668394 (5 μM) for 8 h, followed by removal of NSC305787 and incubation for an additional 20 h.

**i** Another representative images showing H&E staining and immunochemistry staining of mice kidney slices with NRF2, HMOX1, and 4-HNE antibodies. The enlarged view in the lower panel corresponds to the region within the black box in the upper panel for each staining group. Red asterisks indicate large eosinophilic casts during acute kidney injury.

Data and error bars are mean ± SEM, *n =* 3 biologically independent experiments in a-h. *p < 0.05; **p < 0.01; ***p < 0.001; ****p < 0.0001; n.s., not significant. All P values were calculated using a one-way or two-way analysis of variance (ANOVA).

**Supplementary Figure 18** **| ROS-inducing compounds attenuates cystine deprivation-induced ferroptosis.**

**a** Analysis of cell death in HT-1080 cells treated with ML210 (5 μM) combined with DMNQ at the indicated concentrations (5, 10, 15, 20 and 25 μM) for 10.5 h. Dead cells were labeled with Propidium iodide.

**b** Analysis of cell death in HT-1080 cells treated with DMNQ (5, 10, 15, 20 and 25 μM) or Fer-1 (2 μM) under full medium or cystine deprivation for 25 h. Dead cells were labeled with Propidium iodide.

**c** Analysis of cell death in HT-1080 cells treated with RSL3 (2 μM) combined with DMNQ at the indicated concentrations (5, 10, 15, 20 and 25 μM) for 11.5 h. Dead cells were labeled with Propidium iodide.

**d** Analysis of cell death in HT-1080 cells treated with ML210 (0.5 μM) combined with Elesclomol at the indicated concentrations (25, 50,100, 150 and 200 μM) for 8 h. Dead cells were labeled with Propidium iodide.

**e** Analysis of cell death in HT-1080 cells pre-treated with Elesclomol (3, 4.5, 6, 7.5 and 9 μM) for 4 h and then under full medium or cystine deprivation for 22 h. Dead cells were labeled with Propidium iodide.

**f** Analysis of cell death in HT-1080 cells treated with RSL3 (0.5 μM) combined with Elesclomol at the indicated concentrations (25, 50,100, 150 and 200 μM) for 8.5 h. Dead cells were labeled with Propidium iodide.

**g** Analysis of cell death in HT-1080 cells pre-treated with the indicated amount of 3% (W/V) H_2_O_2_ (1, 1.1, 1.2, 1.3 and 1.4 μL 3% H_2_O_2_ add to 1.5 mL GM) for 4 h followed by a combined treatment with ML210 (0.5 μM) treatment for 6 h. Dead cells were labeled with Propidium iodide.

**h** Analysis of cell death in HT-1080 cells pre-treated with the indicated amount of 3% (W/V) H_2_O_2_ (0.25, 0.35, 0.55, 0.65 and 0.75 μL per 1.5 mL GM) for 4 h and then under full medium or cystine deprivation for 21 h. Dead cells were labeled with Propidium iodide.

**i** Analysis of cell death in HT-1080 cells pre-treated with the indicated amount of 3% (W/V) H_2_O_2_ (0.9, 1, 1.1, 1.2 and 1.3 μL 3% H_2_O_2_ add to 1.5 mL GM) for 4.5 h followed by a combined treatment with RSL3 (0.5 μM) treatment for 7 h. Dead cells were labeled with Propidium iodide.

**j** Analysis of cell death in HT-1080 cells pre-treated with the indicated amount of tBOOH (30, 32.5, 35, 37.5 and 40 μM) for 4 h followed by a combined treatment with ML210 (0.5 μM) treatment for 8 h. Dead cells were labeled with Propidium iodide.

**k** Analysis of cell death in HT-1080 cells pre-treated with tBOOH (5, 10, 20, 30 and 40 μM) for 4 h and then under full medium or cystine deprivation for 24 h. Dead cells were labeled with Propidium iodide.

**l** Analysis of cell death in HT-1080 cells pre-treated with the indicated amount of tBOOH (30, 32.5, 35, 37.5 and 40 μM) for 4 h followed by a combined treatment with RSL3 (0.5 μM) treatment for 8 h. Dead cells were labeled with Propidium iodide.

Data and error bars are mean ± SEM, *n =* 3 biologically independent experiments in a-l. *p < 0.05; **p < 0.01; ***p < 0.001; ****p < 0.0001; n.s., not significant. All P values were calculated using a one-way or two-way analysis of variance (ANOVA).

**Supplementary Figure 19** **| ROS-inducing compounds increase the expression of NRF2 relevant genes.**

**a** qPCR analysis of KEAP1 mRNA levels in HT-1080 cells treated with DMNQ (10 μM) for 4 h.

**b** qPCR analysis of KEAP1 mRNA levels in HT-1080 cells treated with tBOOH (30 μM) for 4 h.

**c** qPCR analysis of KEAP1 mRNA levels in HT-1080 cells treated with 3% H_2_O_2_ (1 μL 3% H_2_O_2_ add to 1.5 mL GM) for 4 h.

**d** qPCR analysis of KEAP1 mRNA levels in HT-1080 cells treated with Elesclomol (3 μM) for 4 h.

**e** qPCR analysis of NRF2 mRNA levels in HT-1080 cells treated with DMNQ (10 μM) for 4 h.

**f** qPCR analysis of NRF2 mRNA levels in HT-1080 cells treated with tBOOH (30 μM) for 4 h.

**g** qPCR analysis of NRF2 mRNA levels in HT-1080 cells treated with 3% H_2_O_2_ (1 μL 3% H_2_O_2_ add to 1.5 mL GM) for 4 h.

**h** qPCR analysis of NRF2 mRNA levels in HT-1080 cells treated with Elesclomol (3 μM) for 4 h.

**i** qPCR analysis of SLC7A11 mRNA levels in HT-1080 cells treated with DMNQ (10 μM) for 4 h.

**j** qPCR analysis of SLC7A11 mRNA levels in HT-1080 cells treated with tBOOH (30 μM) for 4 h.

**k** qPCR analysis of SLC7A11 mRNA levels in HT-1080 cells treated with 3% H_2_O_2_ (1 μL 3% H_2_O_2_ add to 1.5 mL GM) for 4 h.

**l** qPCR analysis of SLC7A11 mRNA levels in HT-1080 cells treated with Elesclomol (3 μM) for 4 h.

Data and error bars are mean ± SEM, *n =* 3 biologically independent experiments in a-l. *p < 0.05; **p < 0.01; ***p < 0.001; ****p < 0.0001; n.s., not significant. All P values were calculated using a one-way or two-way analysis of variance (ANOVA).
